# Exploring perspectives of type 2 diabetes prevention program coaches and training delivery staff on e-learning training: a qualitative study

**DOI:** 10.1186/s12909-024-06437-4

**Published:** 2024-12-18

**Authors:** Kaela D. Cranston, Natalie J. Grieve, Mary E. Jung

**Affiliations:** School of Health and Exercise Sciences, University of British Columbia, Okanagan Campus, 1238 Discovery Ave, Kelowna, British Columbia V1V 1V7 Canada

**Keywords:** Technology-enhanced learning, Prediabetic state, Program evaluation, Platform, Interviews, Pragmatism, Feedback

## Abstract

**Background:**

E-learning can be an effective and efficient mode of training healthcare practitioners. E-learning training for diabetes prevention program coaches was designed and developed with input from end users. Insight from those who deliver the training and coaches who have taken the training can provide critical feedback for further refinement of the e-learning training. The purpose of this study was to understand diabetes prevention coaches’ (i.e., those taking the training) and training delivery staffs’ (i.e., those overseeing the training) perspectives of the coach e-learning training. Individuals wishing to become diabetes prevention program coaches were required to complete and pass the e-learning training to become a certified coach.

**Methods:**

A pragmatic paradigm guided the methodology for this study. Semi-structured interviews were conducted with a purposive sample of diabetes prevention program coaches (*n* = 8) and diabetes prevention program training staff (*n* = 3). Interviews were recorded, transcribed verbatim, and analyzed using template analysis. Themes were separately constructed from coach and staff data.

**Results:**

There were seven high order themes constructed from the coach data: (a) training design, (b) “I didn’t know what to expect from the training”, (c) technology usability, (d) learning, (e) coaches’ backgrounds shaped their training experience, (f) support, and (g) coaches valued the training. Two high order themes were constructed from the staff interviews: (a) streamlining the training delivery, and (b) ensuring coaches meet the diabetes prevention program standard.

**Conclusions:**

This study highlights the importance of exploring perspectives of both those receiving and delivering e-learning training to refine content and processes. Qualitatively evaluating the delivery of e-learning training and modifying the training based on the evaluation results can lead to a more acceptable, efficient, and effective e-learning training. Coaches and staff emphasized the benefits of having high-quality online components, and that the brief training promoted gains in knowledge and improvements in skills. Resultswere used to inform modifications to the coach e-learning training for this diabetes prevention program and can be used to inform other healthcare practitioner e-learning trainings.

**Supplementary Information:**

The online version contains supplementary material available at 10.1186/s12909-024-06437-4.

## Background

With the widespread accessibility of the internet and the ramifications of the COVID-19 pandemic, e-learning has emerged as a prominent option for training healthcare practitioners [1, 2]. E-learning provides a flexible and time-efficient method for individuals to receive training [3]. Systematic reviews examining the effects of e-learning in health education demonstrated that learners (i.e., professionals and students in the fields of oncology [4] or nursing [5]) were satisfied with e-learning training and experienced increases in knowledge, skills, confidence, and self-efficacy [4, 5]. The benefits of e-learning training include, but are not limited to, individualized learning, flexibility for learners in terms of geographical location and time, accessing updated resources, and the ability to revisit the training [3, 6]. Challenges associated with e-learning include large up-front financial costs, technology issues, and a lack of communication and feedback between learners and those delivering training [3, 6].

To optimize the effectiveness of e-learning, it is imperative that e-learning training evaluation follows a systematic approach characterized by a well-defined framework and incorporates input from end-users [7]. One such framework is Cook and Ellaway’s [7] technology-enhanced learning (TEL) evaluation framework, which begins with a needs analysis and environmental scan to understand the need for e-learning and the components to be included before developing the e-learning training. Incorporating end-user (i.e., the learners) input into the development process can increase the likelihood that the training components will be appropriate, and the training will be acceptable and effective [8, 9]. This framework comprehensively addresses process and formative evaluation of TEL, which includes e-learning. While using a framework and incorporating end-user feedback can improve the chances of e-learning training being satisfactory and effective, e-learning training improvements can also be made throughout the implementation phase. Evaluating e-learning training is important to judge effectiveness and also provides insightful information on improvements that can be made [7].

### Context

Small Steps for Big Changes (SSBC) is a community-based type 2 diabetes prevention program delivered in fitness and recreation facilities, hereafter referred to as sites, in Canada. Individuals at risk of developing type 2 diabetes meet with a trained SSBC coach (i.e., fitness facility staff member) for six one-on-one sessions over the course of four to six weeks. Coaches use a motivational interviewing (MI)-informed approach [10] to deliver exercise (e.g., gauging exercise intensity) and nutritional (e.g., reducing added sugar and improving carbohydrate choices) information to clients.

SSBC is delivered by fitness facility staff who have received SSBC coach training/certification. The coach e-learning training was developed using the TEL evaluation framework [7] and an integrated knowledge translation (IKT) approach [11–13]. The IKT approach to develop the SSBC e-learning training included focus groups with SSBC coaches who had been trained using the previous in-person version of the SSBC coach training, SSBC research team meetings regarding the goals and needs of the training, and continued input from coaches throughout the development stages. Details about the TEL evaluation framework activities 1–3 (needs analysis, design and development, and usability testing) are published elsewhere [13]. The goal of using the TEL evaluation framework and incorporating input from coaches and the research team was to develop an e-learning training that would be highly satisfactory to users, effectively increase coaches’ knowledge from pre- to post-training, and teach coaches to deliver SSBC sessions to clients with high fidelity.

### The SSBC e-learning training

The e-learning training consists of five components: (1) signing a non-disclosure agreement (NDA) to protect the SSBC research team’s intellectual property; (2) a pre-training knowledge test; (3) seven asynchronous modules covering information on cultural safety and inclusivity, type 2 diabetes information, SSBC session content, and MI; (4) a mock session with an SSBC training delivery staff member; (5) and a post-training knowledge test (using the same questions from the pre-training knowledge test). An online platform (hosted by 3C Institute) houses the modules and a resource centre. The online modules incorporate didactic educational videos, interactive activities (e.g., matching activities), and knowledge checks. The resource centre includes additional information, session guides (i.e., scripts), and video roleplay examples featuring actors portraying scenarios of coaches interacting with clients. Coaches are required to pass both the mock session and post-training knowledge test. Mock sessions were initially conducted on a video conference software customized for SSBC, and were moved to Zoom for an improved user experience (see *Coach theme 3* for more detail). To pass the mock session, coaches are required to demonstrate a client-centred level of MI (assessed by the abbreviated Motivational Interviewing Competency Assessment tool; [14]) while delivering SSBC session 1 content. Coaches are required to receive a minimum score of 70% on the post-training knowledge test. Coaches have unlimited attempts for both the mock session and post-training knowledge test.

Grieve and colleagues [15] demonstrated that SSBC coaches quantitatively rated the training as highly acceptable, and significantly increased program knowledge from pre- to post-training. Further exploration into coaches’ and staffs’ perspectives on the training can offer insight into what is working well and where improvements can be made in the SSBC coach e-learning training and can potentially lead to improved coach and client outcomes. Thus, the purpose of this study was to explore SSBC coaches’ and training delivery staffs’ perspectives of the SSBC coach e-learning training.

## Methods

### Paradigmatic position

This study was guided by a pragmatic paradigm, which prioritizes solving practical problems in the real-world [16, 17]. Pragmatism accepts the use of methods that best suit the research problem under investigation; that is, using methods that are appropriate and practical [17]. In line with the pragmatic paradigm, this study employed a qualitative descriptive methodology because the research question required low-inference interpretation rather than exploring deeper meanings [18].

It is important for transparency to report that all three authors are affiliated with the SSBC research team. All authors’ involvement in SSBC aligns with the pragmatic paradigm, and we assert its necessity for both rich data collection and analytic methods. All three authors are female. The senior author is a professor and the founder of SSBC, and the first and second authors were full-time graduate (i.e., PhD) students working on SSBC under the senior author’s supervision. The second author was also involved in portions of the training delivery as an SSBC training delivery staff member. The first and senior authors developed the SSBC coach e-learning training [13] and are the video narrators within the online training module videos, and therefore, all coach participants were familiar with these two authors prior to partaking in the study. All three authors are committed to improving the coach e-learning training for coaches and staff. Tracy’s [19] eight big-tent criteria and Smith and McGannon’s [20] recommendations for approaching rigour were considered to enhance the research quality using a relativist approach [21]. Specifically, we developed our coding templates using a framework, engaged in self-reflexivity, collected data from different user perspectives (i.e., coaches and delivery staff) for crystallization, reported quotes from multiple participants, considered usefulness of the data, conducted the research in an ethical manner, maintained methodological coherence, and used a critical friend. Additionally, the first author engaged in reflexive journaling throughout the data analysis process to reflect on biases and assumptions.

### Participants and recruitment

At the outset of the study, 52 coaches had completed the SSBC e-learning training, and six training delivery staff were involved in various stages of overseeing the training. Information power [22] is a pragmatic model for appraisal of sample size, and considers study aim (narrow to broad), sample specificity (dense to sparse), theoretical background (applied to none), quality of dialogue (strong to weak), and strategy for analysis (case to cross-case) to determine sample size. Information power values the quality of data to answer a research question (based on the factors previously listed) rather than attempting to calculate sample size. Using information power [22], we aimed to recruit eight SSBC coaches due to the narrow study aim, dense sample specificity, applied theoretical background, a medium-to-strong dialogue quality, and a cross-case analysis. Prior to beginning the e-learning training, all SSBC coaches were asked in an online survey whether they would be interested in being contacted for an interview after completing the training and the training satisfaction survey [15]. Purposive sampling was used to recruit a diverse sample of coaches based on site, rural or urban setting, and self-reported sex. Recruiting a diverse sample of coaches aligned with our pragmatic approach, aiming to capture a spectrum of perspectives. Participants were invited to join the study via email. Four additional coaches were invited to participate in interviews but did not respond to the invitation email.

We aimed to recruit three SSBC training delivery staff based on information power [22]. SSBC training delivery staff were involved in some or all of overseeing the coach training process, facilitating mock sessions, and coding mock sessions. They were recruited approximately two years after the launch of the SSBC coach e-learning training. Staff were purposively recruited to ensure we gained insight from those involved in various aspects of training delivery (e.g., oversight, communication, mock session delivery, mock session coding) and those involved in the delivery of the training at the start of implementation and at two years post-implementation to capture the iterative changes that have been made over time. All participants were aware that the purpose of this study was to gather feedback on the SSBC coach e-learning training.

### Interviews

One-on-one semi-structured interviews were conducted between April 2022 to September 2023 over Zoom (version 5.16.0) and lasted approximately one hour each (21 min to 1 h 35 min), with no one present other than the interviewer and interviewee. Interviews were recorded using Zoom and transcribed using Otter.ai (version 3.50). Participants did not receive the interview questions ahead of time. All participants selected their own pseudonyms to ensure anonymity. The interview guides for SSBC coaches and SSBC training delivery staff were different. Both interview guides were developed to evaluate all aspects of the coach training and training delivery. Interview questions were created to ask about challenges, satisfaction, proposed modifications, and general experiences. The SSBC coach interview guide asked questions centred on coaches’ experiences going through the entire training process, individual components of the training, delivering SSBC to clients, and the value of the training on their professional or personal growth. The interview guide for SSBC training delivery staff covered topics including the training delivery process, sustainability of the training process, and communication with coaches. Interview guides were piloted with two research assistants who had taken the SSBC coach e-learning training. Changes included minor wording modifications for comprehension and the addition of some prompts. Final interview guides can be found in Supplementary file 1.

### Coach interviews

At the outset of the study, SSBC coaches were interviewed three months after receiving SSBC coach certification so that they had experience using their training with SSBC clients. After conducting six coach interviews, we added a new interview timepoint immediately post-certification so that we could gain insights from coaches on the training immediately after certification to prevent hindsight bias. The original interview guide was modified for the new timepoint to remove questions about coaches’ experiences delivering SSBC to clients. One coach was interviewed at both the new timepoint and the original three-month timepoint to capture new perspectives based on experience delivering SSBC to clients. The second author conducted the SSBC coach interviews to reduce power imbalances so coaches would feel comfortable sharing both positive and negative feedback on the SSBC coach training.

### Staff interviews

SSBC training delivery staff were interviewed by the first author two years after implementation of the coach e-learning training to ensure that there was diversity in coaches and organizations involved in the coach e-learning training. Delivery staff were informed that their supervisor (the senior author) would not be involved in the interview and would not be told which of their staff participated in interviews due to perceived or real conflicts of interest. All three staff members held different roles in the training delivery. Interviewing each staff member was critical to gain diverse insights.

### Data analysis

The first author reviewed all Otter.ai transcripts and corrected any transcription errors. All transcriptions were then imported to NVivo (version 12) for analysis. Template analysis [23, 24] was used to analyze all transcript data. Separate templates were used for the coach and staff interview analyses. Participants were offered the opportunity to review their cleaned transcripts, but none accepted this offer.

### Coach interviews

The initial template for coach interviews was informed by the Technology Enhanced Learning Framework Quality Domains [7]. The first and second authors reviewed the nine quality domains, discussed their applicability to the study objective and interview guide questions, discussed ideas that the quality domains missed that were included in the interview guide, and created the first version of the coach interview coding template. This first version consisted of five main codes, each with two to six subcodes. Supplementary File 2 shows the first version of the coach interview coding template. The first and second authors each used the first version of the coach template to independently code one transcript from different coaches. The first and second authors then met to discuss inadequacies in the template and modifications required (e.g., insertion or deletion of codes, changing scope, changing classification). The first author then coded all coach transcripts using the updated version of the coach template, continuing to make changes as needed. In total, the first author went through each coach transcript three times, re-coding as necessary with the continually updated coach template. The first author consulted the second author about template changes when uncertainties arose. After three rounds of coding, the first author deemed the coach template as final. The second author acted as a critical friend, reviewing the final themes, and discussing with the first author to encourage reflexivity [20] and achieve descriptive and interpretive validity [18].

### Staff interviews

The first version of the staff coding template was developed by inductively coding the three staff interviews. Codes were added to the template if they addressed the study objective. After coding each staff interview once, the first author examined the codes, grouped similar codes together, and created a template. The template was used to recode the staff interview transcripts, with modifications occurring as needed. After three total rounds of coding, the template was finalized. The senior author acted as a critical friend for staff interviews, with the same responsibilities as the critical friend for the coach interviews.

### Reporting

The highest order themes are reported in the *Results* section of this paper, with an overview of some of the lower order themes incorporated.

## Results

### Participants

Eight coaches and three SSBC training delivery staff participated in this study. Half of the SSBC coaches self-identified as female, with the other half self-identifying as male. Most coaches were delivering SSBC in an urban fitness facility. On average, SSBC training delivery staff were involved in training delivery for 20 months. Additional participant demographics for coaches and staff can be found in Table 1.

**Table 1:** Participant demographics

	Coaches	Staff
	*N*	*N*
Age (years)		
18–24	1	1
25–34	4	2
35–44	1	0
45+	2	0
Sex		
Female	4	2
Male	4	1
Gender		
Man or primarily masculine	4	1
Woman or primarily feminine	4	2
Work status		
Working full-time (30 h/week or more)	2	1
Working part-time (below 30 h/week) and student	4	2
Student	1	0
Retired	1	0

### SSBC coach interview findings

**Fig. 1:**
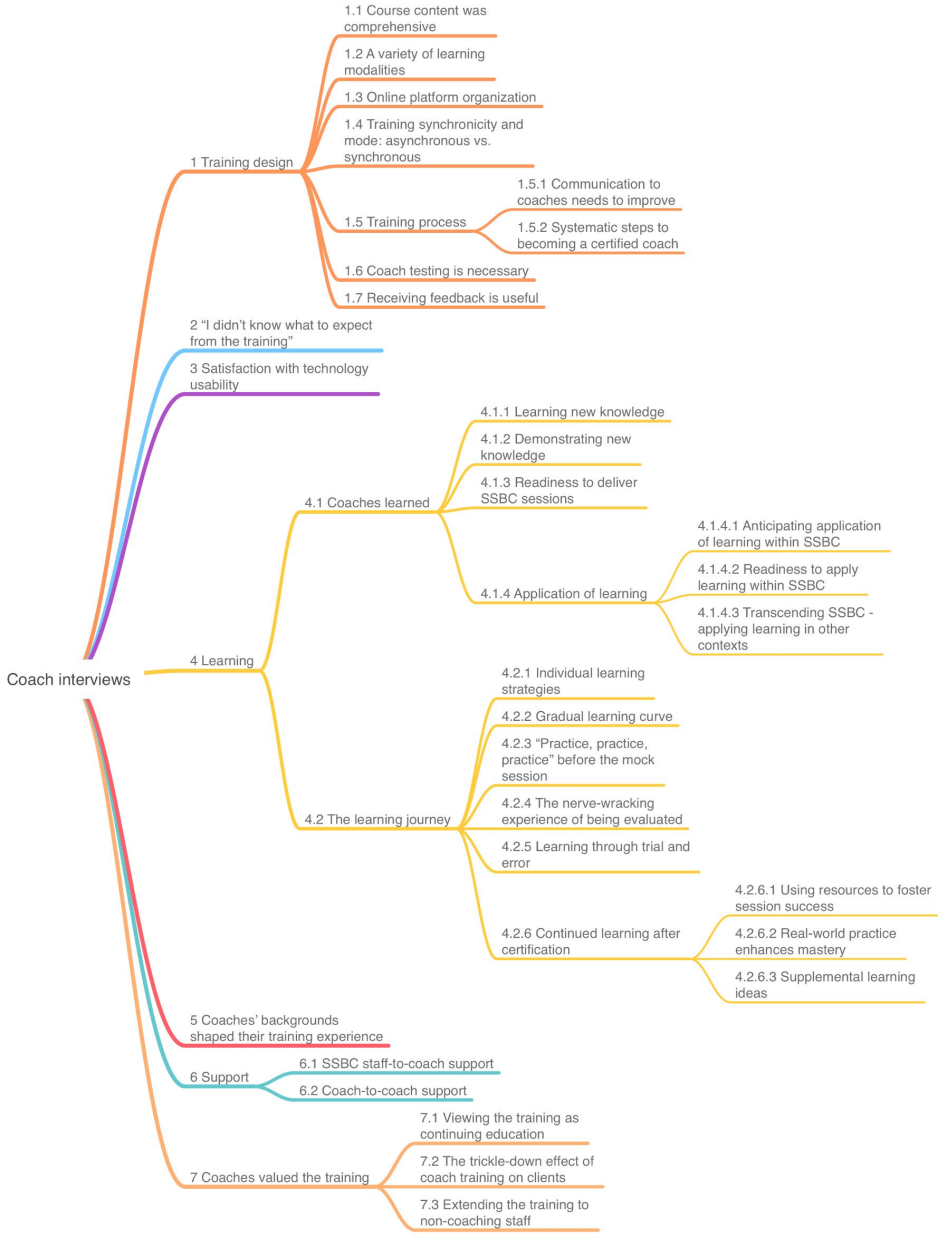
Final template for coach interview analysis

There were seven high order themes and 32 lower order themes constructed from the SSBC coach interviews. See Fig. 1 for the final SSBC coach thematic template. High order themes are described below with exemplar quotes in Table 2.

**Table 2:** Coach interview themes and exemplar quotes

Themes	Exemplar quotes
1 Training design	n/a
1.1 Course content was comprehensive	“There’s quite a bit of training and it’s very in-depth training.” - Tina
1.2 A variety of learning modalities	“I would say [the mock session] was really helpful because, yeah, it kind of gets the kinks out before you actually do it [with real clients].” - Deborah
1.3 Online platform organization	“[The resource centre was] labelled well enough so you know what you’re looking for or looking at.” - Tina
1.4 Training synchronicity and mode: asynchronous vs. synchronous	“[I] learn better when a human talks to me instead of [watching a] video.” - Lebron
1.5 Training process	n/a
1.5.1 Communication to coaches needs to improve	“[It would be nice to have] a simple graph that says, you know, first step is a pre-test, and then the seven modules with quizzes.” - Marisol
1.5.2 Systematic steps to becoming a certified coach	“[The training followed] a competency-based approach, which…was enlightening…and a complete process because there was a pretest for the knowledge, then it went into the modules, and then it went to the mock…So theoretically, do you know the information, now you can apply it and implement…and then a final test for knowledge.” - Marisol
1.6 Coach testing is necessary	“I obviously took [the mock session] pretty seriously and wanted to pass it.” - Marisol
1.7 Receiving feedback is useful	“It was good because there were some things that I missed [in the mock session] and that was pointed out to me. So, it was good, once again, going into my first session to remind myself not to forget certain items.” - Mike
2 “I didn’t know what to expect from the training”	“[I knew that I’d learn about] prediabetes and helping clients to know how to exercise and to eat healthier.” - Sally
3 Satisfaction with technology usability	“Everything worked as it should, which is good because I’m pretty technologically challenged. So didn’t have any issues.” - Tina
4 Learning	n/a
4.1 Coaches learned	n/a
4.1.1 Learning new knowledge	“[I learned that] it comes down to more obviously client-centred…more or less kind of motivate them.” - Paul
4.1.2 Demonstrating new knowledge	“[I learned] the skills on like coaching things, like how to concisely teach the talk test, and how to concisely talk about carbohydrates and sugar. And only like giving the actual information that is wanted from the client, so not word vomiting a bunch of information to people, and so just being like, oh, what do they want…kind of listening.” - Deborah
4.1.3 Readiness to deliver SSBC sessions	“The actual modules and the training process, whether it was like the pretest and the modules right through the mock session, I felt competent at the end – that I can start to go and deliver to the clients.” - Marisol
4.1.4 Application of learning	n/a
4.1.4.1 Anticipating application of learning within SSBC	“I have to be careful of the biases, right. So like, I can walk into a gym and I’m not intimidated, right. But I think for some people that will be their first experience, right? Or it could be very intimidating. So I think there’s some things that we have in our, you know, in our own toolbox that will apply and make us more aware.” - Marisol
4.1.4.2 Readiness to apply learning within SSBC	“I think the most important…the things that I can see that I changed about the way I talk to clients, that I do differently in SSBC is listen more. So ask them to tell more about things instead of just providing them [with information].” - Sally
4.1.4.3 Transcending SSBC – applying learning in other contexts	“I do find the motivational interviewing can actually be very helpful in the personal training.” - Tina
4.2 The learning journey	n/a
4.2.1 Individual learning strategies	“I took notes, and I’m old school, so I write things down. Right. And I [used] cue cards as well, for when I was preparing for the mock, and also for delivering the sessions with the client.” - Marisol
4.2.2 Gradual learning curve	“I assumed [the training] was going to be easier than it was. So it was a learning curve.” - Mike
4.2.3 “Practice, practice, practice” before the mock session	“I started to practice [MI] around the house with my husband and friends and family.” - Marisol
4.2.4 The nerve-wracking experience of being evaluated	“[The SSBC staff] guided me through in the beginning of the session so that I knew what to, like, expect, which helped me a lot to relax a little bit and focus more on what I had to ask and accomplish.” - Ryan
4.2.5 Learning through trial and error	“[My colleague failed the mock session.] So I asked, what happened? What didn’t you do?…[They] showed me the printouts and how to go over it…It definitely helped that my co-worker did [the training] before me.” - Lebron
4.2.6 Continued learning after certification	n/a
4.2.6.1 Using resources to foster session success	“With my first two clients, I mentioned to them that they were my first two clients. So I had the [guide] in front of me. So I was making sure that I [had] all the information down. And I even told them like, I don’t want to be like robotic, but I’m just like learning.” - Ryan
4.2.6.2 Real-world practice enhances mastery	“I think [with] every client, I’m a little bit better at [using MI].” - Tina
4.2.6.3 Supplemental learning ideas	“It’s easy to swing back to our old, I guess, ways of doing things as a coach, so training calls [after being certified] would help.” - Marisol
5 Coaches’ backgrounds shaped their training experience	“The motivational interview, that is something completely new for me. I’m way better than before the training, but I still think I have a lot to improve on…And for the specifically about diabetes. I already knew those information. So that thing wasn’t new for me. And it’s a good part because then I can focus my efforts in being better about how to coach the session and not necessarily about to know the technical information.” - Sally
6 Support	n/a
6.1 SSBC staff-to-coach support	“The feedback was really nice and encouraging and gave helpful tips and stuff.” - Deborah
6.2 Coach-to-coach support	“I printed [the resources] off for all my coaches that are coming…so they have a successful first, first interview.” - Tina
7 Coaches valued the training	n/a
7.1 Viewing the training as continuing education	“Especially for personal trainers, I think we do learn a lot about exercising, but we don’t learn about how to talk to people. So it’s not just telling them that they should exercise and that they will get the importance of it. So it is very important for us to know more [about] how to talk to people, and the SSBC training helps us to do this.” - Sally
7.2 The trickle-down effect of coach training on clients	“As I get better at [MI], I think it is helping [my clients] take a bigger role in how they’re coming up with the ideas themselves. And I think that in the long run, they will be more successful than me telling them what to do.” - Tina
7.3 Extending the training to non-coaching staff	“I think it would be really good for like all the managers to do motivational interviewing, even customer service and aquatics. As well as the inclusivity, I think it’s important. I don’t think every department has to learn about diabetes prevention, but I think just some of the like, skills are transferrable.” - Deborah

n/a: no exemplar quotes for themes containing lower order themes

#### Training design

Coaches shared their perspectives on the overall design of the training and were satisfied with the training. The comprehensiveness of the training and the thoroughness of the resource centre were highlighted, although there were mixed views on the resource centre layout. Coaches had varied opinions about which learning modalities were most useful, ranging from the mock session to the resources, to the knowledge checks and interactive activities. Half of the coaches in this study had a strong desire for there to be in-person training components. Specifically, coaches suggested that the mock session would be an ideal component to be conducted in-person, although they acknowledged that incorporating in-person and additional synchronous components could be inconvenient or impractical.

Generally, coaches requested better communication around the details of the training process and some steps, specifically regarding what to expect for the mock session and knowledge tests. Coaches perceived all the steps within the training to be logical and important, as they started with learning information and then worked on applying the information in the mock session. Coaches understood that the testing components were necessary so that the SSBC staff could ensure coaches knew the material well enough to become certified coaches. Seven of the eight coaches highlighted the importance and utility of the mock session, specifically that they learned from the feedback on both their mock session fails and passes.

#### “I didn’t know what to expect from the training”

Most coaches did not know what to expect from the training before they began. Coaches had a very general idea about what they would learn. Some coaches learned about what to expect from other coaches at their site, which was deemed helpful.

#### Technology usability

Two main technology platforms were used for the SSBC coach e-learning training: the online platform that housed the online modules, resources, and pre- and post-training knowledge tests; and a program-specific video conferencing platform to host the mock session video calls. Coaches shared their perspectives on the ease of use and technical difficulties associated with these platforms. Overall, coaches were extremely satisfied with the simplicity and professionalism of the online platform with the modules. While coaches had no issues with the online platform, one coach suggested that adding the option to display subtitles could be useful for some coaches. When it came to the video conferencing software, some coaches experienced technical difficulties related to joining the call, not being able to access all features of the software (e.g., accessing the space for writing client notes during the session and accessing the session checklist within the platform). After the switch to using Zoom instead of the original video conferencing software, coaches did not experience any technical issues.

#### Learning

Throughout the interviews, all coaches discussed learning from the training and their learning journey through the training. With regard to learning, coaches discussed learning new knowledge from the training modules. They talked about being able to take what they learned and practice before certification, with the mock session being particularly beneficial. Coaches also shared their perspectives on applying everything they learned from the training into practice. For those coaches who had not yet started to deliver SSBC to clients (interview timepoint 1), they talked about their anticipated application of learning within SSBC. Coaches who had already begun delivering SSBC to clients (interview timepoint 2) discussed both the ways they were applying their learning within SSBC sessions and in other contexts as well, such as personal training.

Coaches had various approaches to completing the training and viewed the mock session as an important piece to the learning journey. Most of the coaches highlighted the importance of practicing prior to the mock session to increase the likelihood of passing the mock on their first attempt. Some coaches shared that they practiced their skills with other SSBC coaches or their family members before their mock session. While coaches did not view the mock session as a negative training component, many discussed the nerves associated with going into a situation where they would be evaluated. Some coaches described feeling nervous going into the mock session, but relaxing as they began talking to the SSBC staff delivering the mock session. Four coaches talked about learning both training information and more about the training process from failing the mock session or from a colleague failing the mock session. An unanticipated theme that we constructed was around the idea that coaches continued to learn after receiving their SSBC coach certification. They reported to continue their learning with the resource documents and through delivering real-world SSBC sessions. Additionally, coaches provided suggestions for continued learning beyond certification.

#### Coaches’ backgrounds shaped their training experience

While every coach learned from the training, some coaches had backgrounds that affected the ease of their learning journey. English was two of the coaches’ second language, which made the training more challenging for them. Some coaches had previously learned MI or had backgrounds in health and exercise, so those components of the training served as more of a refresher.

#### Support

Coaches received support throughout the training from the SSBC staff as well as from other coaches. Coaches frequently reported feeling most supported by the SSBC staff during the mock session and in the feedback that the SSBC staff provided for coaches following the mock session. In the instances where coaches going through the training had co-workers who had already completed the training, they all spoke about how the certified coach supported them throughout their training process. This support helped coaches prepare for mock sessions, and understand which resources were useful. On the flip side, coaches also shared ideas for how they could receive more support throughout the training process. Some coaches suggested that the training design could be enhanced by adding in more touchpoints between SSBC staff and coaches.

#### Coaches valued the training

The final theme that we constructed from the coach interview data centred around the notion that coaches saw value in the SSBC coach training. One idea that coaches spoke about was that they saw value in the coach training as well as the SSBC program in general. Coaches also described that this training was valuable for their careers, that their training would positively affect the clients that they work with, and that other employees within their sites could benefit from pieces of the SSBC coach e-learning training.

### SSBC training delivery staff interview findings

**Fig. 2:**
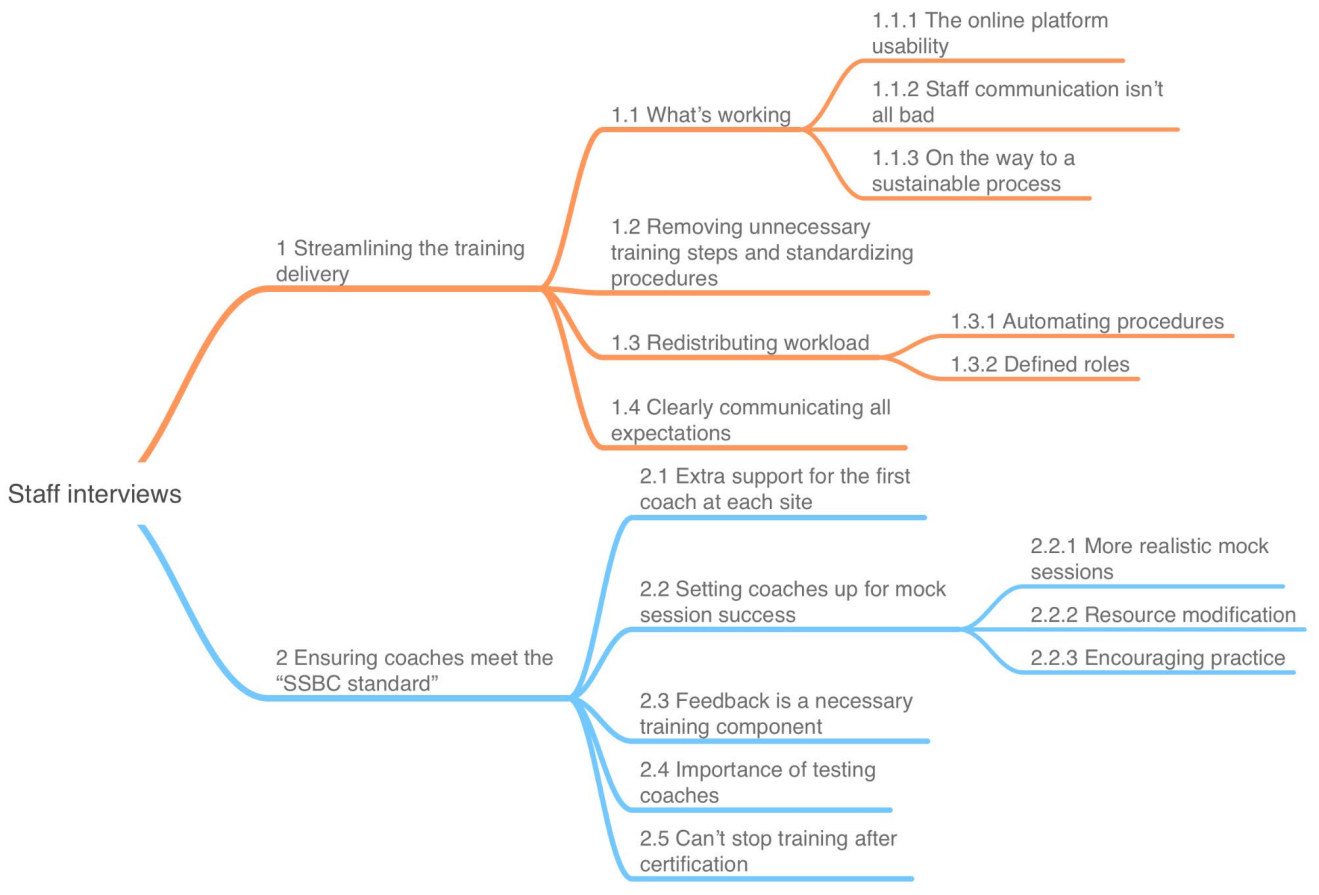
Final template for staff interview analysis

We constructed two high order themes and 17 lower order themes from the SSBC training delivery staff interviews (see Fig. 2). Descriptions of each high order theme are below, with exemplar quotes found in Table 3.

**Table 3:** Staff interview themes and exemplar quotes

Themes	Exemplar quote
1 Streamlining the training delivery	n/a
1.1 What’s working	n/a
1.1.1 The online platform usability	“I really like the way that the platform kind of takes control of the training for most of it. And then there’s only really three instances where I need to be in contact with them. And the like, there seems to be a kind of flexibility around what notifications I can send from the platform, which is quite useful.” - John
1.1.2 Staff communication isn’t all bad	“[The staff are] supporting [coaches] throughout the process…even though we’re getting a lot of emails, John’s always responding. So they should feel hopefully supported.” - Jennifer
1.1.3 On the way to a sustainable process	“[The online training] process has allowed us to train a lot of coaches really quickly with less resources [than in-person training], and I think that’s a huge win.” - Jennifer
1.2 Removing unnecessary training steps and standardizing procedures	“And something I’ve been doing is to just whenever I release a notification [from the platform], I also email [site leads], particularly at the start because if it gets redirected to their spam folder then they can move it and [their inbox] should accept the rest of the emails that come. But I have wondered if it’s maybe worth like, at the start of new sites, asking site leads or senior management to whitelist the emails from [the online platform] so they’re never blocked.” - John
1.3 Redistributing workload	n/a
1.3.1 Automating procedures	“It’s a bit like administratively laborious to have the NDA separate when it could be included in the [online platform] login page.” - John
1.3.2 Defined roles	“[There is a] back and forth between a coach and the like three different people on our team….it feels like there’s so many spots where something can get messed up.” - Sophie
1.4 Clearly communicating all expectations	“Maybe [providing] just a bit more information about what the mock session is…and possibly like, the fact that it takes 45 min.” - John
2. Ensuring coaches meet the “SSBC standard”	n/a
2.1 Extra support for the first coach at each site	“The mock session can feel a bit intimidating, particularly for people who are either the first in their site or just haven’t had contact with anyone else in the site. And it’s come up [in conversations with coaches] that support around the mock would be valuable.” - John
2.2 Setting coaches up for mock session success	n/a
2.2.1 More realistic mock sessions	“95% of the mock sessions I code is the coach with the script open on their desk, reading it line for line for line for line. And if we want our coaches to be delivering SSBC at a client-centred way, having conversations their client, reading a script isn’t doing that.” - Sophie
2.2.2 Resource modification	“I just sometimes think that the script, like the script for like, the sessions are actually like, confusing a little. I feel like there’s a lot of information in them so then [coaches]…we’re trying to make it as like simple as possible by giving them all this info, but it’s actually really overwhelming.” - Sophie
2.2.3 Encouraging practice	“Adding another mock session is not sustainable. I think encouraging potential practice though, with especially at the site…Maybe we upload like a mock client script of some sort [to the resource centre], and they can like practice with someone at their [site] who’s also training and they can switch roles.” - Jennifer
2.3 Feedback is a necessary training component	“I think the feedback is almost equally [important as doing the mock session]…I write them feedback so that if they were to implement all of those pieces [from the feedback], they would be delivering SSBC [to our standards].” - Sophie
2.4 Importance of testing coaches	“[Coaches] learn a lot from the modules. I think a mock session gives them a chance to practice their skills and get feedback.” - Jennifer
2.5 Can’t stop training after certification	“It think it could be beneficial for a yearly, two-hour Zoom booster, where there’s a 40-minute refresher – what MI is, maybe some mock scenarios with [the training delivery staff]. And [coaches] can tune in, watch it, we record it, or send it to them. And maybe there’s some practice breakout rooms, something like that.” - Jennifer

n/a: no exemplar quotes for themes containing lower order themes

#### Streamlining the training delivery

SSBC staff discussed several ways to streamline the training delivery process. While the training delivery process was less resource-intensive than in-person delivery, there were still back-end tasks that staff had to complete. Staff highlighted what was currently working well with the training (i.e., the online platform usability, some of the communication, and that TEL is a sustainable training method). They also discussed ways to improve the training, such as standardizing procedures, identifying and eliminating unnecessary steps, improving communication to coaches, and utilizing automation and technology.

#### Ensuring coaches meet the “SSBC standard”

Staff highlighted the importance of ensuring that certified SSBC coaches were reaching and maintaining a level of proficiency that met the standards of the SSBC research team. The SSBC research team asserts that SSBC coaches deliver the program with knowledge of the SSBC program content and at a client-centred level of MI. Staff shared the perspective that changes made to the training should ensure coaches continue to reach or exceed those standards. Suggestions included providing extra support for the first coach at each site and improving the mock session process by changing the format of some resources, making the mock session more realistic, and encouraging coaches to practice the mock session with another coach or their site lead before completing the formal mock session with an SSBC staff member. Additionally, staff viewed the mock session feedback as a critical step in the coach training process. The staff noted that evaluating the coaches’ skills and knowledge was important for the coaches to learn and to ensure that all coaches met the SSBC standard before delivering SSBC to real-world clients. Finally, all three SSBC staff believed that the training should not stop after coaches are certified. The staff did not think that additional components should be added to the training process for coaches to become certified, but did discuss ways to help the coaches learn after certification and ensure coaches were maintaining their skills and knowledge. Suggestions included incorporating a community of practice or forum for coaches to connect with and learn from other coaches; refresher courses to maintain certification; and regular meetings with coaches at each site led by site leads.

## Discussion

E-learning is an efficient and effective way to deliver training to large numbers of people spread across large geographical areas [3, 6]. Understanding the perspectives of e-learning training users is important to further refine e-learning training to enhance effectiveness, acceptability, and sustainability. The purpose of this study was to understand SSBC coaches’ and staffs’ perspectives on the SSBC coach e-learning training. Several themes were constructed in this study to capture coaches’ and staffs’ various perspectives. There were seven high order themes constructed from the coach interviews: (a) training design, (b) “I didn’t know what to expect from the training”, (c), technology usability, (d) learning, (e) coaches’ background shaped their training experience, (f) support, and (g) coaches valued the training; and two high order themes constructed from the staff interviews: (a) streamlining the training delivery and (b) ensuring coaches meet the “SSBC standard”.

The online modules and resource centre were favourably viewed by all coaches and staff. Technology issues are often cited as one of the downsides to e-learning [3], however, it is possible that the high up-front cost associated with the online platform for this training helped to avoid technology issues. Staff suggested additional applications for the online platform to further alleviate their workload. Automating staff tasks could enhance the cost-effectiveness of the training because once they are implemented, these components would require minimal staff attention and oversight.

Coaches and staff all saw high value in the mock session training component as a means for learning and ensuring coaches were at the SSBC standard of delivery before delivering the program to clients at sites. The value placed on the opportunity to apply the learning from the asynchronous modules is echoed in a study by Jones and colleagues [25], where a group of physiotherapists using e-learning found a mock scenario of high value. The nervousness that coaches felt before engaging in the mock session could potentially be minimized by staff suggestions of clearer communication of expectations and better preparing coaches for the mock session. Some coaches will likely feel nervous before the mock session regardless of changes because the purpose of the mock session is to evaluate the coach’s MI skills to ensure they meet the SSBC standard to pass the training. These feelings of nervousness related to a mock scenario were also identified in the study of physiotherapists [25]. Coaches’ desire for more synchronous and in-person training components, specifically surrounding the mock session (see *coach subtheme 1.4 training synchronicity and mode*) needs to be balanced with sustainability as the training scales up and reaches more coaches. The staff recommendation of promoting coaches practice for the mock session with peers and the site lead, has the possibility of striking the balance between giving coaches further practice and maintaining a sustainable training process. It is possible that as e-learning continues to become more widespread, coaches will have less desire for in-person and synchronous components due to their familiarity and confidence in e-learning training designs and their ability to learn through e-learning training.

The majority of the SSBC coach e-learning training design and development efforts were focussed on the process coaches would go through to become certified [13]. Less attention was paid to the staff delivery of the e-learning training, which was reflected in several themes. Standardization of staff procedures, including clearer communication, needs to be highly prioritized to enhance both the coaches’ and staffs’ training experiences. It is possible that less attention was given to the staff experience due to a scarcity of research on e-learning training design and development [7].

Evaluating user experience for both those taking and delivering e-learning training is important to capture nuances associated with training design. Despite the training design still needing further improvements, it is promising that coaches highlighted that they learned from the SSBC coach e-learning training. Coaches shared what they learned and discussed their ability to practice and apply their gained knowledge and skills both within their SSBC sessions and in other contexts. The training design allowed coaches to follow a systematic training process, while also utilizing their own learning strategies and approaches. One strength to e-learning over in-person training is that learners can utilize their own learning strategies and can rewatch e-learning modules at their convenience. SSBC coaches highlighted this as a strength, and this has also been cited as a strength in research involving healthcare students [26]. Incorporating input from SSBC coaches during the design and development phases of the SSBC coach e-learning training and working with experts in e-learning likely played a role in attenuating different learning styles.

Our findings support Cook and Ellaway’s [7] sentiment of the importance of assessing participant experience and satisfaction. Despite thoroughly incorporating SSBC coach input and feedback during the design and development processes of the SSBC coach e-learning training, there are still many areas for improvement within the training. The two main user groups for this e-learning training were SSBC coaches and the SSBC staff facilitating the delivery of the training. Findings suggest that investing both time and resources into usable and high-quality technology may be worthwhile to enhance all users’ experience. The use of technology that goes beyond PowerPoint slides and voice-over may lead to improved learner engagement as well as reducing staff burden.

Based on the feedback provided in this study from both coaches and training delivery staff, the SSBC coach e-learning training was modified. Modifications included automating more training steps to reduce staff burden, delivery staff providing more and clearer communication with coaches regarding training expectations, and modifying and adding coach resources to the resource centre. With more fitness and recreation facilities being onboarded to deliver SSBC, the coach e-learning training is currently scaling up. The modifications that were made to the training gives us confidence that the timing of the training scale-up is appropriate.

The results of this paper have led us to develop the following recommendations for individuals and groups developing their own TEL within the health context:

1. Invest in resources (i.e., time, money) during the development stages of e-learning because learners value high quality training.
2. Provide learners the opportunity to gain knowledge and then practice skills.
3. Providing feedback on learners’ progress is an additional mode of learning.
4. Ensure clear communication to learners is provided throughout the training process.
5. If e-learning includes asynchronous components, suggesting that learners study or practice together could be beneficial.
6. Standardize and automate as many steps of the e-learning process as possible to ensure an efficient process for learners and staff.

### Limitations

The sample of coaches and staff was fairly homogenous with regard to age, which could have implications regarding technology literacy. As SSBC continues to scale-up nationally and globally, there will be greater diversity in coach and staff demographics, and future research may be needed to further explore the experiences of those belonging to demographic groups not represented in this study. Secondly, this study was specific to the SSBC coach e-learning training, which limits generalizability of study results. However, we attempted to structure the coding templates and results in a manner that other e-learning training developers or evaluators could learn from these results. Additionally, coach interviews were predominantly conducted approximately three months after certification, which could limit their memory of how long they took to complete training and could have affected their perspective on the training. While we attempted to minimize power dynamics between interviewers and participants, it is possible that participants perceived power differentials, which could have affected the data. Furthermore, as with all research, it is possible that participants were not honest or had reservations about providing negative feedback about the SSBC coach e-learning training. We attempted to reduce the chances of this by asking participants for all positive and negative comments and assuring participants that negative feedback would not affect their roles in SSBC. We believe this helped with participant honesty, as we did receive feedback from participants on areas for improvement within the e-learning training.

### Future directions

Coaches and staff discussed that coaches learned from the SSBC coach e-learning training and that there was a positive trickle-down effect for clients. In line with Cook and Ellaway’s TEL evaluation framework [7], future research must assess the learning outcomes (e.g., Kirkpatrick levels 2, 3, and 4) from this training, and a cost estimate of this training should be conducted to fully understand the sustainability.

## Conclusions

This study highlights the perspectives and experiences of coaches and staff as they interacted with the SSBC coach e-learning training. Understanding coach and staff perspectives is important given that they are the individuals delivering and receiving the training. Results demonstrated that coaches and staff valued a high-quality online platform, the importance of developing training with both coaches and staff in mind, and that incorporating several learning modalities and modes could cater to different learning styles and backgrounds. E-learning developers can consider the results from this study when creating their own e-learning platforms to enhance user satisfaction. This study demonstrates that continued evaluation beyond the design and development of e-learning training can provide insightful information which could lead to more effective, efficient, and acceptable e-learning training iterations.

## Electronic supplementary material

Below is the link to the electronic supplementary material.

Supplementary Material 1

Supplementary Material 2

Supplementary Material 3

## Data Availability

The dataset analyzed during the current study are available from the corresponding author on reasonable request.

## Abbreviations

IKT: Integrated knowledge translation
MI: Motivational interviewing
NDA: Non-disclosure agreement
TEL: Technology-enhanced learning
