# Supplementary material for: Exploring perspectives of type 2 diabetes prevention program coaches and training delivery staff on e-learning training: a qualitative study
Source: BMC Med Educ. 2024 Dec 18;24:1469. doi: 10.1186/s12909-024-06437-4 (PMC11653984; doi:10.1186/s12909-024-06437-4)
Supplement: Supplementary file 1 — Supplementary Material 1 [file 12909_2024_6437_MOESM1_ESM.docx]

**Coach Interview Guide**

- What made you want to become an SSBC coach?
- How many clients have you coached in the SSBC program?
- Tell me about your overall experience as a coach for the SSBC program.
- Can you tell me about the training you went through to become an SSBC coach?
- What did the training involve?
  - *Probe for insight around each step of training to become an SSBC coach - this will anchor them to think about how long this entire process takes.*

The next set of questions will ask about the entire training process. ‘The entire training process’, refers to the training process from when you got access to the training to when you received the certificate. This entire training process consists of the online modules, the mock session, and the post-training survey.

- Can you please talk about your experience with the entire training process?
- How satisfied were you with the entire training process?
- How long did the training process took you?
  - Duration from getting access to receiving certificate.
  - How many hours to you think that you spent on the training? Modules? Preparing for mock session?
- Did you feel you had enough training/were well-prepared by the time you had your mock session?
  - If no, what would’ve helped?
- Did you feel you had enough training/were well-prepared by the time you had your first session with a client?
- Thinking about the entire training process, what aspect(s) did you find most useful?
- Thinking about the entire training process, what aspects did you find least useful?
- Did you feel that the entire training program increased your skills and knowledge to be a coach? Explain.
  - What skills, knowledge?
- What information/skills/topics should be modified in the training to better prepare SSBC coaches for their clients?
- Thinking about the entire training process, what aspects do you think need to be refined when working with SSBC clients?
  - Is there anything that comes up in sessions with SSBC clients that you feel unprepared to deal with?
  - What parts of delivering SSBC confuses you that we could clarify in the training?
- What were your expectations from the training before you started it?
  - In what ways did the training meet your expectations and/or needs?
  - In what ways did the training fail to meet your expectations and/or needs?
- What recommendations do you have to improve the overall training for future SSBC coaches?
- If we were to offer ongoing support for coaches after they’ve been certified, what would you like this to look like? (e.g., videos to watch, real-time practice)

The following questions will ask you about the online training platform. You can talk about any aspects of the online training platform from the seven modules and resource centre to the content included and the usability of the platform and technology.

- Tell me about your overall experience with the online training platform.
- What skills/knowledge did you gain from the online training modules?
- What feedback do you have (positive or negative) about the online training platform?
- What do you think could be modified or done differently on the online training platform?
- Did you visit the resource centre?
  - If yes, in what way(s) did you use the resource centre? How often?
  - If no, why not?
- What, if anything, do you think could be added to the resource centre?
- Can you tell me about your experience using the training website (e.g., navigating it, technological difficulties)?

The following questions will ask you about your mock session with the delivery staff. You can talk about any aspects of the mock session from your preparation for the mock session, the mock session itself, and the feedback that you received after your mock session.

- Tell me about your experience with the mock session after you completed the online modules.
- What skills/knowledge did you gain from the mock session?
- What feedback do you have (positive or negative) about the mock session?
- What do you think could be modified or done differently for the mock session?
- Did you read the feedback that was provided to you after your mock session?
  - If yes, what did you take away from the feedback? How did you use the feedback?
- [If they mention that they did the mock call more than once] What differences occurred between your preparation and the mock session itself from your first to second mock session?

The following questions will ask you about the post-training survey that you completed after passing the mock session.

- Did you prepare for the post-training survey?
  - If yes, how?
- After passing your mock session, you received a link to the post-training. Are/were you aware that you had to pass this in order to become a certified SSBC coach?

The following questions will ask you about your experiences with delivering the SSBC program to clients after you completed and passed the training.

- What do you feel is working well for you when you are coaching SSBC clients?
- What challenges have you experienced with delivering SSBC?
- What skills have you used from the training in your delivery of SSBC?
- What else, if anything, would help you to increase your confidence in facilitating the SSBC program?
- Now that you’ve taken the SSBC coach training and have been delivering SSBC to clients, what do you think was missing from the training?
- How do you feel your MI skills are impacting the outcomes of your SSBC clients?
- How do you think the use of your MI skills is affecting your relationship with your SSBC clients?
- What helps you use the MI skills when you are coaching your clients? (e.g., notes, practicing at home, practicing with colleagues, revisiting modules or the resource centre)
- What MI skills did you learn in the training that you use the most during your sessions with a client?
- What would help you to maintain your MI skills over time?

The following questions will ask you about whether you’ve used anything from the SSBC training in your work outside of SSBC.

- Have you ever taken any other training that covered topics similar to those in the SSBC coach training (e.g., MI; diabetes; equity, diversity, and inclusion)?
  - If yes, what training and what topics?
- Have you used what you learned in the training beyond your role as an SSBC coach?
  - If yes, how?
- Do you think that people working in other positions in your facility would benefit from taking any components of the SSBC coach training?
  - If so, what positions, what components, and why?
- What value would you place on this training?
- Is there anything else you’d like to discuss that we didn’t talk about today?

**Delivery Staff Interview Guide**

- Can you please describe your role in the coach training and certification process?
- Overall, how is the training process going?
  - What is working well?
  - What is not working well?
- What improvements can be made to the training process?
- Please describe communication between delivery staff and coaches and/or managers, and how you think that’s going.
  - What’s going well?
  - What is not going well?
  - What improvements could be made?
- What, if any, feedback have you received from coaches or managers about how the training process is going? This can include feedback on changes that we’ve already made or changes that we haven’t made and can be positive or negative feedback.
- Can you think of any additional resources we should add to the resource centre?
- What, if anything, should be changed (added, removed, modified) in the online modules?
- Do you think that all components of the training process (e.g., NDA, pre-training questionnaire, modules, mock, post-training questionnaire) are necessary?
  - Why or why not?
- Do you think the mock session is a necessary component of the training?
  - If yes
    - Why?
    - What do you think it adds to the training?
    - What could be done differently (added, removed, modified) to improve this portion of the training?
    - Is our mock session process (one mock session with feedback) enough?
  - If no
    - Why not?
    - Is there something else that should be done instead?
- Do you think the pre- and post-training surveys are a necessary component of the training?
  - If yes
    - Why?
    - What do you think it adds to the training?
    - What could be done differently (added, removed, modified) to improve this portion of the training?
  - If no
    - Why not?
    - Is there something else that should be done instead?
- What support do delivery staff provide to coaches regarding the training?
  - Thinking about sustainability as the program expands, what do you envision support from delivery staff to coaches going through the training looking like?
- What supports do managers/site leads provide to coaches regarding the training?
  - Thinking about sustainability as the program expands, what do you see support from managers to coaches going through the training looking like?
- What supports do coaches provide each other regarding the training?
  - Thinking about sustainability as the program expands, what do you see support from trained coaches to coaches going through the training looking like?
- What other supports could we provide coaches going through the training?
- Can you discuss the sustainability of the online training?
  - How can the training be changed to be more sustainable?
  - What’s going well in terms of making this a sustainable process?
  - What is hindering sustainability?
  - Suggestions for improving sustainability.
- Per coach, how many hours are you spending on getting them trained (from the beginning to the end)?
  - How many hours are others spending on this?
- Is there a need for additional training beyond this training process?
- Do you have any additional thoughts or ideas that regarding the training that we haven’t yet talked about?
