# Supplementary material for: Exploring perspectives of type 2 diabetes prevention program coaches and training delivery staff on e-learning training: a qualitative study
Source: BMC Med Educ. 2024 Dec 18;24:1469. doi: 10.1186/s12909-024-06437-4 (PMC11653984; doi:10.1186/s12909-024-06437-4)
Supplement: Supplementary file 2 — Supplementary Material 2 [file 12909_2024_6437_MOESM2_ESM.docx]

SSBC Online Coach Training Coding Template

1. Process
   1. Communication
   2. Individual approach to training
      1. Duration
      2. Learning strategies (e.g., taking notes)
      3. Practice (e.g., before mock or first client)
   3. Order of training components/steps (e.g., module > mock > post-training survey)
2. Training/course design
   1. Content
   2. Organization of course (e.g., order of modules)
   3. Feedback (mock, post-training survey, knowledge checks)
   4. Synchronicity/mode
   5. Modalities of learning (e.g., videos, interactives)
3. Support
   1. From DPRG
   2. From other coaches
   3. Recommendations for other coaches
4. Learning
   1. SSBC competencies/skills
   2. SSBC-related knowledge
   3. Application of knowledge
      1. Within SSBC
      2. Beyond SSBC
5. Value place on training
   1. Recommend for other staff
   2. Extended/continuing education
