# Supplementary material for: Exploring perspectives of type 2 diabetes prevention program coaches and training delivery staff on e-learning training: a qualitative study
Source: BMC Med Educ. 2024 Dec 18;24:1469. doi: 10.1186/s12909-024-06437-4 (PMC11653984; doi:10.1186/s12909-024-06437-4)
Supplement: Supplementary file 3 — Supplementary Material 3 [file 12909_2024_6437_MOESM3_ESM.docx]

**Exploring perspectives of type 2 diabetes prevention program coaches and training delivery staff on e-learning training: A qualitative study**

Kaela D Cranston^a^, Natalie J Grieve^a^, Mary E Jung^a^

^a^ School of Health and Exercise Sciences, University of British Columbia, Okanagan Campus, 3333 University Way, Kelowna, Canada, V1V 1V7

**Corresponding author:** Mary E Jung, School of Health and Exercise Sciences, University of British Columbia, Okanagan Campus, 1238 Discovery Ave, Kelowna, British Columbia, Canada, V1V 1V7

Email: [mary.jung@ubc.ca](mailto:mary.jung@ubc.ca)

Tel: +1 250 807 9670

**Abstract**

*Background:* E-learning can be an effective and efficient mode of training healthcare practitioners. E-learning training for diabetes prevention program coaches was designed and developed with input from end users. Insight from those who deliver the training and coaches who have taken the training can provide critical feedback for further refinement of the e-learning training. The purpose of this study was to understand diabetes prevention coaches’ (i.e., those taking the training) and training delivery staffs’ (i.e., those overseeing the training) perspectives of the coach e-learning training. Individuals wishing to become diabetes prevention program coaches were required to complete and pass the e-learning training to become a certified coach. *Methods:* A pragmatic paradigm guided the methodology for this study. Semi-structured interviews were conducted with a purposive sample of diabetes prevention program coaches (n=8) and diabetes prevention program training staff (n=3). Interviews were recorded, transcribed verbatim, and analyzed using template analysis. High and lower order themes were separately constructed from coach and staff data. *Results:* There were seven high order themes constructed from the coach data: a) training design, b) “I didn’t know what to expect from the training”, c) technology usability, d) learning, e) coaches’ backgrounds shaped their training experience, f) support, and g) coaches valued the training. Two high order themes were constructed from the staff interviews: a) streamlining the training delivery, and b) ensuring coaches meet the diabetes prevention program standard. *Conclusions:* This study highlights the importance of exploring perspectives of both those receiving and delivering e-learning training to refine content and processes. Qualitatively evaluating the delivery of e-learning training and modifying the training based on the evaluation results can lead to a more acceptable, efficient, and effective e-learning training. Coaches and staff emphasized the benefits of having high-quality online components, and that the brief training promoted gains in knowledge and improvements in skills. Results can be used to inform modifications to the coach e-learning training for this diabetes prevention program and to inform other healthcare practitioner e-learning trainings.

Keywords: technology-enhanced learning, prediabetic state, program evaluation, platform, interviews, pragmatism, feedback

**Background**

With the widespread accessibility of the internet and the ramifications of the COVID-19 pandemic, e-learning has emerged as a prominent option for training healthcare practitioners [1,2]. E-learning provides a flexible and time-efficient method for individuals to receive training [3]. Systematic reviews examining the effects of e-learning in health education demonstrated that learners (i.e., professionals and students in the fields of oncology [4] or nursing [5]) were satisfied with e-learning training and experience increases in knowledge, skills, confidence, and self-efficacy [4,5]. The benefits of e-learning training include, but are not limited to, individualized learning, flexibility for learners in terms of geographical location and time, accessing updated resources, and the ability to revisit the training [3,6]. Challenges associated with e-learning include large up-front financial costs, technology issues, and a lack of communication and feedback between learners and those delivering training [3,6].

To optimize the effectiveness of e-learning, it is imperative that e-learning training evaluation follows a systematic approach characterized by a well-defined framework and incorporates input from end-users [7]. One such framework is Cook and Ellaway’s [7] technology-enhanced learning (TEL) evaluation framework, which begins with a needs analysis and environmental scan to understand the need for e-learning and the components to be included before developing the e-learning training. Incorporating end-user (i.e., the learners) input into the development process can increase the likelihood that the training components will be appropriate, and the training will be acceptable and effective [8,9]. This framework comprehensively addresses process and formative evaluation of TEL, which includes e-learning. While using a framework and incorporating end-user feedback can improve the chances of e-learning training being satisfactory and effective, e-learning training improvements can also be made throughout the implementation phase. Evaluating e-learning training is important to judge effectiveness and also provides insightful information on improvements that can be made [7].

**Context**

[PROGRAM NAME BLINDED FOR REVIEW] is a community-based type 2 diabetes prevention program delivered in fitness and recreation facilities, hereafter referred to as sites. Individuals at risk of developing type 2 diabetes meet with a trained [PROGRAM NAME BLINDED FOR REVIEW] coach (i.e., fitness facility staff member) for six one-on-one sessions over the course of four to six weeks. Coaches use a motivational-interviewing (MI)-informed approach [10] to deliver exercise (e.g., gauging exercise intensity) and nutritional information (e.g., reducing added sugar and improving carbohydrate choices) to clients.

[PROGRAM NAME BLINDED FOR REVIEW] is delivered by fitness facility staff who have received [PROGRAM NAME BLINDED FOR REVIEW] coach training/certification. The coach e-learning training was developed using the TEL evaluation framework [7] and an integrated knowledge translation (IKT) approach [11–13]. The IKT approach to develop the [PROGRAM NAME BLINDED FOR REVIEW] e-learning training included focus groups with [PROGRAM NAME BLINDED FOR REVIEW] coaches who had been trained using the previous in-person version of the [PROGRAM NAME BLINDED FOR REVIEW] coach training, [PROGRAM NAME BLINDED FOR REVIEW] research team meetings regarding the goals and needs of the training, and continued input from coaches throughout the development stages. For more details about the TEL evaluation framework activities 1-3 (needs analysis, design and development, and usability testing), please see [REFERENCE BLINDED FOR REVIEW] [13]. The goal of using the TEL evaluation framework and incorporating input from coaches and the research team was to develop an e-learning training that would be highly satisfactory to users, effectively increase coaches’ knowledge from pre- to post-training, and teach coaches to deliver [PROGRAM NAME BLINDED FOR REVIEW] sessions to clients with high fidelity.

**The [PROGRAM NAME BLINDED FOR REVIEW] e-learning training**

The e-learning training consists of five components: 1) signing a non-disclosure agreement (NDA) to protect the [PROGRAM NAME BLINDED FOR REVIEW] research team’s intellectual property; 2) a pre-training knowledge test; 3) seven asynchronous modules covering information on cultural safety and inclusivity, type 2 diabetes information, [PROGRAM NAME BLINDED FOR REVIEW] session content, and MI; 4) a mock session with an [PROGRAM NAME BLINDED FOR REVIEW] training delivery staff member; 5) and a post-training knowledge test (using the same questions from the pre-training knowledge test). An online platform (hosted by 3C Institute) houses the modules and a resource centre. The online modules incorporate didactic educational videos, interactive activities (e.g., matching activities), and knowledge checks. The resource centre includes additional information, session guides (i.e., scripts), and video roleplay examples featuring actors portraying scenarios of coaches interacting with clients. Coaches are required to pass both the mock session and post-training knowledge test. Mock sessions were initially conducted on a video conference software customized for [PROGRAM NAME BLINDED FOR REVIEW], and were moved to Zoom for an improved user experience (see *Coach theme 3* for more detail). To pass the mock session, coaches are required to demonstrate a client-centred level of MI (assessed by the abbreviated Motivational Interviewing Competency Assessment tool; [14]) while delivering [PROGRAM NAME BLINDED FOR REVIEW] session 1 content. Coaches are required to receive a minimum score of 70% on the post-training knowledge test. Coaches had unlimited attempts for both the mock session and post-training knowledge test.

[REFERENCE BLINDED FOR REVIEW] [15] demonstrated that [PROGRAM NAME BLINDED FOR REVIEW] coaches quantitatively rated the training as highly acceptable, and significantly increased program knowledge from pre- to post-training. Further exploration into coaches’ and staffs’ perspectives on the training can offer insight into what is working well and where improvements can be made in the [PROGRAM NAME BLINDED FOR REVIEW] coach e-learning training and can potentially lead to improved coach and client outcomes. Thus, the purpose of this study was to explore [PROGRAM NAME BLINDED FOR REVIEW] coaches’ and training delivery staffs’ perspectives of the [PROGRAM NAME BLINDED FOR REVIEW] coach e-learning training.

**Methods**

**Paradigmatic position**

This study was guided by a pragmatic paradigm, which prioritizes solving practical problems in the real-world [16,17]. Pragmatism accepts the use of methods that best suit the research problem under investigation; that is, using methods that are appropriate and practical [17]. In line with the pragmatic paradigm, this study employed a qualitative descriptive methodology because the research question required low-inference interpretation rather than exploring deeper meanings [18].

It is important for transparency to report that all three authors are affiliated with the [PROGRAM NAME BLINDED FOR REVIEW] research team. All authors’ involvement in [PROGRAM NAME BLINDED FOR REVIEW] aligns with the pragmatic paradigm, and we assert its necessity for both rich data collection and analytic methods. All three authors are female. The senior author is a professor and the founder of [PROGRAM NAME BLINDED FOR REVIEW], and the first and second authors were full-time graduate (i.e., PhD) students working on [PROGRAM NAME BLINDED FOR REVIEW] under the senior author’s supervision. The second author was also involved in portions of the training delivery as an [PROGRAM NAME BLINDED FOR REVIEW] training delivery staff member. The first and senior authors developed the [PROGRAM NAME BLINDED FOR REVIEW] coach e-learning training [13] and are the video narrators within the online training module videos, and therefore, all coach participants were familiar with these two authors prior to partaking in the study. All three authors are committed to improving the coach e-learning training for coaches and staff. Tracy’s [19] eight big-tent criteria and Smith and McGannon’s [20] recommendations for approaching rigour were considered to enhance the research quality using a relativist approach [21]. Specifically, we developed our coding templates using a framework, engaged in self-reflexivity, collected data from different user perspectives (i.e., coaches and delivery staff) for crystallization, reported quotes from multiple participants, considered usefulness of the data, conducted the research in an ethical manner, maintained methodological coherence, and used a critical friend. Additionally, the first author engaged in reflexive journaling throughout the data analysis process to reflect on biases and assumptions.

**Participants and recruitment**

At the outset of the study, 52 coaches had completed the [PROGRAM NAME BLINDED FOR REVIEW] e-learning training, and six training delivery staff were involved in various stages of overseeing the training. Information power [22] is a pragmatic model for appraisal of sample size, and considers study aim (narrow to broad), sample specificity (dense to sparse), theoretical background (applied to none), quality of dialogue (strong to weak), and strategy for analysis (case to cross-case) to determine sample size. Information power values the quality of data to answer a research question (based on the factors previously listed) rather than attempting to calculate sample size. Using information power [22], we aimed to recruit eight [PROGRAM NAME BLINDED FOR REVIEW] coaches due to the narrow study aim, dense sample specificity, applied theoretical background, a medium-to-strong dialogue quality, and a cross-case analysis. Prior to beginning the e-learning training, all [PROGRAM NAME BLINDED FOR REVIEW] coaches were asked in an online survey whether they would be interested in being contacted for an interview after completing the training and the training satisfaction survey [15]. Purposive sampling was used to recruit a diverse sample of coaches based on site, rural or urban setting, and self-reported sex. Recruiting a diverse sample of coaches aligned with our pragmatic approach, aiming to capture a spectrum of perspectives. Participants were invited to join the study via email. Four coaches were invited to participate in interviews but did not respond to the invitation email.

We aimed to recruit three [PROGRAM NAME BLINDED FOR REVIEW] training delivery staff based on information power [22]. [PROGRAM NAME BLINDED FOR REVIEW] training delivery staff were involved in some or all of overseeing the coach training process, facilitating mock sessions, and coding mock sessions. They were recruited approximately two years after the launch of the [PROGRAM NAME BLINDED FOR REVIEW] coach e-learning training. Staff were purposively recruited to ensure we gained staff insight from those involved in various aspects of training delivery (e.g., oversight, communication, mock session delivery, mock session coding) and those involved in the delivery of the training at the start of implementation and at two years post-implementation to capture the iterative changes that have been made over time. All participants were aware that the purpose of this study was to gather feedback on the [PROGRAM NAME BLINDED FOR REVIEW] coach e-learning training.

**Interviews**

One-on-one semi-structured interviews were conducted between April 2022 to September 2023 over Zoom (version 5.16.0) and lasted approximately one hour each (21 minutes to 1 hour 35 minutes), with no one present other than the interviewer and interviewee. Interviews were recorded using Zoom and transcribed using Otter.ai (version 3.50). Participants did not receive the interview questions ahead of time. All participants selected their own pseudonyms to ensure anonymity. The interview guides for [PROGRAM NAME BLINDED FOR REVIEW] coaches and [PROGRAM NAME BLINDED FOR REVIEW] training delivery staff were different. Both interview guides were developed to evaluate all aspects of the coach training and training delivery. Interview questions were created to ask about challenges, satisfaction, proposed modifications, and general experiences. The [PROGRAM NAME BLINDED FOR REVIEW] coach interview guide asked questions centred on coaches’ experiences going through the entire training process, individual components of the training, delivering [PROGRAM NAME BLINDED FOR REVIEW] to clients, and the value of the training to their professional or personal growth. The interview guide for [PROGRAM NAME BLINDED FOR REVIEW] training delivery staff covered topics including the training delivery process, sustainability of the training process, and communication with coaches. Interview guides were piloted with two research assistants who had taken the [PROGRAM NAME BLINDED FOR REVIEW] coach e-learning training. Changes included minor wording changes for comprehension and the addition of some prompts. Final interview guides can be found in Supplementary file 2.

*Coach interviews*

At the outset of the study, [PROGRAM NAME BLINDED FOR REVIEW] coaches were interviewed three months after receiving [PROGRAM NAME BLINDED FOR REVIEW] coach certification so that they had experience using their training with [PROGRAM NAME BLINDED FOR REVIEW] clients. After conducting six coach interviews, we added a new interview timepoint immediately post-certification so that we could gain insights from coaches on the training immediately after certification to prevent hindsight bias. The original interview guide was modified for the new timepoint to remove questions about coaches’ experiences delivering SSBC to clients. One coach was interviewed at both the new timepoint and the original three-month timepoint to capture new perspectives based on experience delivering [PROGRAM NAME BLINDED FOR REVIEW] to clients. The second author conducted the [PROGRAM NAME BLINDED FOR REVIEW] coach interviews to reduce power imbalances so coaches would feel comfortable sharing both positive and negative feedback on the [PROGRAM NAME BLINDED FOR REVIEW] coach training.

*Staff interviews*

[PROGRAM NAME BLINDED FOR REVIEW] training delivery staff were interviewed by the first author two years after implementation of the coach e-learning training to ensure that there was diversity in coaches and organizations involved in the coach e-learning training. Delivery staff were informed that their supervisor (the senior author) would not be involved in the interview and would not be told which of their staff participated in interviews due to perceived or real conflicts of interest. All three staff members held different roles in the training delivery. Interviewing each staff member was critical to gain diverse insights.

**Data analysis**

The senior author reviewed all Otter.ai transcripts and corrected any transcription errors. All transcriptions were then imported to NVivo (version 12) for analysis. Template analysis [23,24] was used to analyze all transcript data. Separate templates were used for the coach and staff interview analyses. Participants were offered the opportunity to review their cleaned transcripts, but none accepted this offer.

*Coach interviews*

The initial template for coach interviews was informed by the Technology Enhanced Learning Framework Quality Domains [7]. The first and second authors reviewed the nine quality domains, discussed their applicability to the study objective and interview guide questions, discussed ideas that the quality domains missed that were included in the interview guide, and created the first version of the coach interview coding template. This first version consisted of five main codes, each with two to six subcodes. Supplementary File 1 shows the first version of the coach interview coding template. The first and second authors each used the first version of the coach template to independently code one transcript from different coaches. The first and second authors then met to discuss inadequacies in the template and modifications required (e.g., insertion or deletion of codes, changing scope, changing classification). The first author then coded all coach transcripts using the updated version of the coach template, continuing to make changes as needed. In total, the first author went through each coach transcript three times, re-coding as necessary with the continually updated coach template. The first author consulted the second author about template changes when uncertainties arose. After three rounds of coding, the first author deemed the coach template as final. The second author acted as a critical friend, reviewing the final themes, and discussing with the first author to encourage reflexivity [20] and achieve descriptive and interpretive validity [18].
*Staff interviews*

The first version of the staff coding template was developed by inductively coding the three staff interviews. Codes were added to the template if they addressed the study objective. After coding each staff interview once, the first author examined the codes, grouped similar codes together, and created a template. The template was used to recode the staff interview transcripts, with modifications occurring as needed. After three total rounds of coding, the template was finalized. The senior author acted as a critical friend for staff interviews, with the same responsibilities as the critical friend for the coach interviews.

*Reporting*

The highest order themes are reported in the *Results* section of this paper, with an overview of some of the lower order themes incorporated.

**Results**

**Participants**

Eight coaches and three [PROGRAM NAME BLINDED FOR REVIEW] training delivery staff participated in this study. Half of the [PROGRAM NAME BLINDED FOR REVIEW] coaches self-identified as female, with the other half self-identifying as male. Most coaches were delivering [PROGRAM NAME BLINDED FOR REVIEW] in an urban fitness facility. On average, [PROGRAM NAME BLINDED FOR REVIEW] training delivery staff have been involved in training delivery for 20 months. Additional participant demographics for coaches and staff can be found in Table 1.

Table 1. Participant demographics

|  | Coaches | Staff |
| --- | --- | --- |
|  | N | N |
| Age (years) |  |  |
| 18-24 | 1 | 1 |
| 25-34 | 4 | 2 |
| 35-44 | 1 | 0 |
| 45+ | 2 | 0 |
| Sex |  |  |
| Female | 4 | 2 |
| Male | 4 | 1 |
| Gender |  |  |
| Man or primarily masculine | 4 | 1 |
| Woman or primarily feminine | 4 | 2 |
| Work status |  |  |
| Working full-time (30 hours/week or more) | 2 | 1 |
| Working part-time (below 30 hours/week) and student | 4 | 2 |
| Student | 1 | 0 |
| Retired | 1 | 0 |

**[PROGRAM NAME BLINDED FOR REVIEW] coach interview findings**

There were seven high order themes and 32 lower order themes constructed from the [PROGRAM NAME BLINDED FOR REVIEW] coach interviews, see Figure 1 for the final [PROGRAM NAME BLINDED FOR REVIEW] coach thematic template. High order themes are described below with exemplar quotes in Table 2.


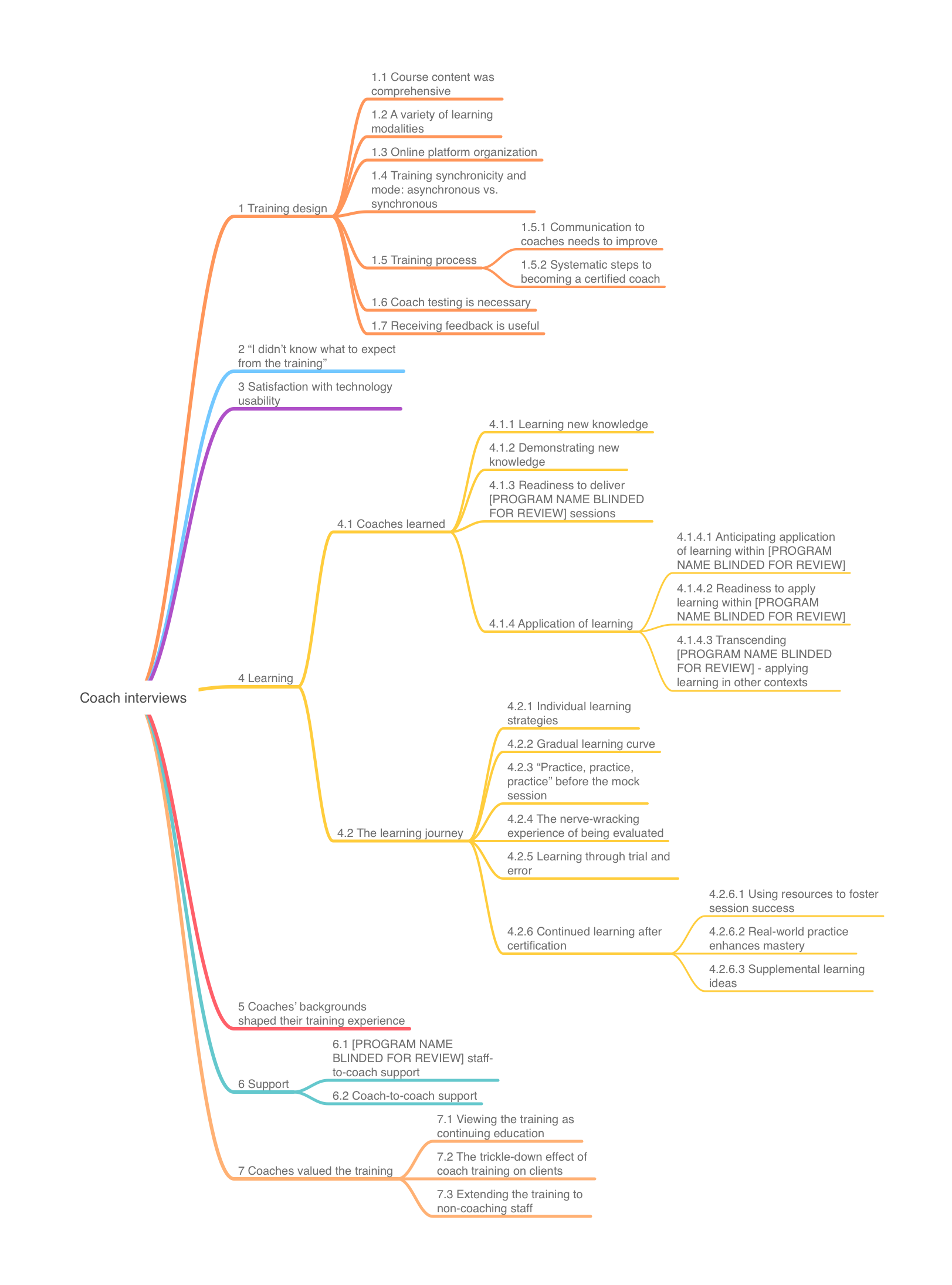


Figure 1. Final template for coach interview analysis

1. Training design

Coaches shared their perspectives on the overall design of the training and were satisfied with the training. The comprehensiveness of the training and the thoroughness of the resource centre were highlighted, although there were mixed views on the resource centre layout. Coaches had varied opinions about which learning modalities were most useful, ranging from the mock session to the resources, to the knowledge checks and interactive activities. Half of the coaches in this study had a strong desire for there to be in-person training components. Specifically, coaches suggested that the mock session would be an ideal component to be conducted in-person, although they acknowledged that incorporating in-person and additional synchronous components could be inconvenient or impractical.

Generally, coaches requested better communication around the details of the training process and specific steps, specifically regarding what to expect for the mock session and knowledge tests. Coaches perceived all the steps within the training to be logical and important, as they started with learning information and then worked on applying the information in the mock session. Coaches understood that the testing components were necessary so that the [PROGRAM NAME BLINDED FOR REVIEW] staff could ensure coaches knew the material well enough to become certified coaches. Seven of the eight coaches highlighted the importance and utility of the mock session, specifically that they learned from the feedback on both their mock session fails and passes.

1. “I didn’t know what to expect from the training”

Most coaches did not know what to expect from the training before they began. Coaches had a very general idea about what they would learn. Some coaches learned about what to expect from other coaches at their site, which was deemed helpful.

1. Technology usability

Two main technology platforms were used for the [PROGRAM NAME BLINDED FOR REVIEW] coach e-learning training: the online platform that housed the online modules, resources, and pre- and post-training knowledge tests; and a program-specific video conferencing platform to host the mock session video calls. Coaches shared their perspectives on the ease of use and technical difficulties associated with these platforms. Overall, coaches were extremely satisfied with the simplicity and professionalism of the online platform with the modules. While coaches had no issues with the online platform, one coach suggested that adding the option to display subtitles could be useful for some coaches. When it came to the video conferencing software, some coaches experienced technical difficulties related to joining the call, not being able to access all features of the software (e.g., accessing the space for writing client notes during the session and accessing the session checklist within the platform). After the switch to using Zoom instead of the original video conferencing software, coaches did not experience any technical issues.

1. Learning

Throughout the interviews, all coaches discussed learning from the training and their learning journey through the training. With regard to learning, coaches discussed learning new knowledge from the training modules. They talked about being able to take what they learned and practice before certification, with the mock session being particularly beneficial. Coaches also shared their perspectives on applying everything they learned from the training into practice. For those coaches who had not yet started to deliver [PROGRAM NAME BLINDED FOR REVIEW] to clients (interview timepoint 1), they talked about their anticipated application of learning within [PROGRAM NAME BLINDED FOR REVIEW]. Coaches who had already begun delivering [PROGRAM NAME BLINDED FOR REVIEW] to clients (interview timepoint 2) discussed both the ways they were applying their learning within [PROGRAM NAME BLINDED FOR REVIEW] sessions and in other contexts as well, such as personal training.

Coaches had various approaches to completing the training and viewed the mock session as an important piece to the learning journey. Most of the coaches highlighted the importance of practicing prior to the mock session to increase the likelihood of passing the mock on their first attempt. Some coaches shared that they practiced their skills with other [PROGRAM NAME BLINDED FOR REVIEW] coaches or their family members before their mock session. While coaches did not view the mock session as a negative training component, many discussed the nerves associated with going into a situation where they would be evaluated. Some coaches described feeling nervous going into the mock session, but relaxing as they began talking to the [PROGRAM NAME BLINDED FOR REVIEW] staff running the mock session. Four coaches talked about learning both training information and more about the training process from failing the mock session or from a colleague failing the mock session. An unanticipated theme that we constructed was around the idea that coaches continued to learn after receiving their [PROGRAM NAME BLINDED FOR REVIEW] coach certification. They reported to continue their learning with the resource documents and through delivering real-world [PROGRAM NAME BLINDED FOR REVIEW] sessions. Additionally, coaches provided suggestions for continued learning beyond certification.

5. Coaches’ backgrounds shaped their training experience

While every coach learned from the training, some coaches had backgrounds that affected the ease of their learning journey. English was two of the coaches’ second language, which made the training more challenging for them. Some coaches had previously learned MI or had backgrounds in health and exercise, so those components of the training served as more of a refresher.

6. Support

Coaches received support throughout the training from the [PROGRAM NAME BLINDED FOR REVIEW] staff as well as from other coaches. Coaches frequently reported feeling most supported by the [PROGRAM NAME BLINDED FOR REVIEW] staff during the mock session and in the feedback that the [PROGRAM NAME BLINDED FOR REVIEW] staff provided for coaches following the mock session. In the instances where coaches going through the training had co-workers who had already completed the training, they all spoke about how the certified coach supported them throughout their training process. This support helped coaches prepare for mock sessions, and understand which resources were useful. On the flip side, coaches also shared ideas for how they could receive more support throughout the training process. Some coaches suggested that the training design could be enhanced by adding in more touchpoints between [PROGRAM NAME BLINDED FOR REVIEW] staff and coaches.

7. Coaches valued the training

The final theme that we constructed from the coach interview data centred around the notion that coaches saw value in the [PROGRAM NAME BLINDED FOR REVIEW] coach training. One idea that coaches spoke about was that they saw value in the coach training as well as the [PROGRAM NAME BLINDED FOR REVIEW] program in general. Coaches also described that this training was valuable for their careers, that their training would positively affect the clients that they work with, and that other employees within their sites could benefit from pieces of the [PROGRAM NAME BLINDED FOR REVIEW] coach e-learning training.

--INSERT TABLE 2 HERE--

**[PROGRAM NAME BLINDED FOR REVIEW] training delivery staff interview findings**

We constructed two high order themes and 17 lower order themes from the [PROGRAM NAME BLINDED FOR REVIEW] training delivery staff interviews (see Figure 2). Descriptions of each high order theme are below, with exemplar quotes found in Table 3.


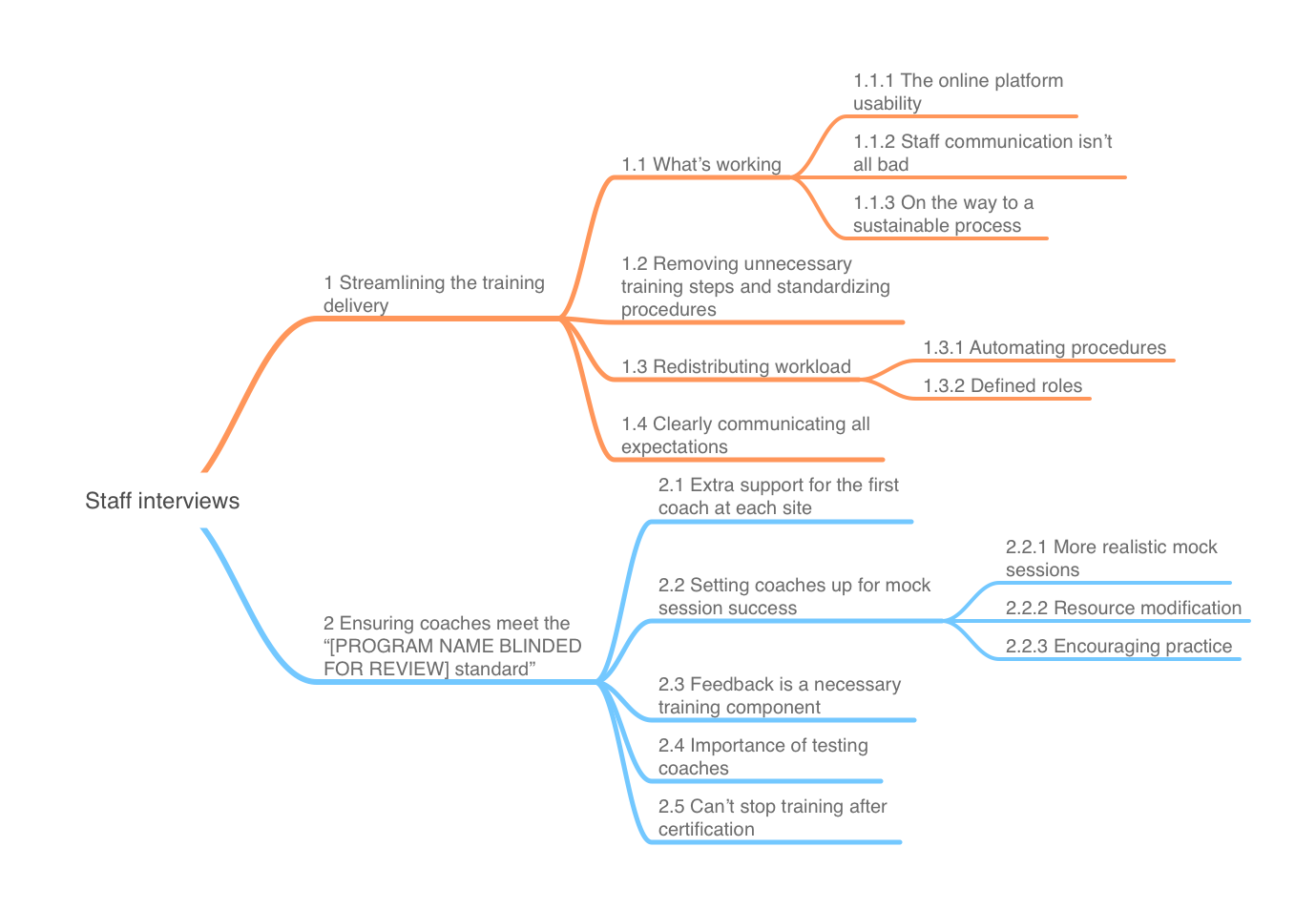


Figure 2. Final template for staff interview analysis

1. Streamlining the training delivery

[PROGRAM NAME BLINDED FOR REVIEW] staff discussed several ways to streamline the training delivery process. While the training delivery process was less resource-intensive than in-person delivery, there were still back-end tasks that staff had to complete. Staff highlighted what is currently working well with the training (i.e., the online platform usability, some of the communication, and that TEL is a sustainable training method). They also discussed ways to improve the training, such as standardizing procedures, identifying and eliminating unnecessary steps, improving communication to coaches, and utilizing automation and technology.

2. Ensuring coaches meet the “[PROGRAM NAME BLINDED FOR REVIEW] standard”

Staff highlighted the importance of ensuring that certified [PROGRAM NAME BLINDED FOR REVIEW] coaches were reaching and maintaining a level of proficiency that met the standards of the [PROGRAM NAME BLINDED FOR REVIEW] research team. The [PROGRAM NAME BLINDED FOR REVIEW] research team asserts that [PROGRAM NAME BLINDED FOR REVIEW] coaches deliver the program with knowledge of the [PROGRAM NAME BLINDED FOR REVIEW] program content and at a client-centred level of MI. Staff shared the perspective that changes made to the training should ensure coaches continue to reach or exceed those standards. Suggestions included providing extra support for the first coach at each site and improving the mock session process by changing the format of some resources, making the mock session more realistic, and encouraging coaches to practice the mock session with another coach or their site lead before completing the formal mock session with an [PROGRAM NAME BLINDED FOR REVIEW] staff member. Additionally, staff viewed the mock session feedback as a critical step in the coach training process. The staff noted that evaluating the coaches’ skills and knowledge was important for the coaches to learn and to ensure that all coaches met the [PROGRAM NAME BLINDED FOR REVIEW] standard before delivering [PROGRAM NAME BLINDED FOR REVIEW] to real-world clients. Finally, all three [PROGRAM NAME BLINDED FOR REVIEW] staff believed that the training should not stop after coaches are certified. The staff did not think that additional components should be added to the training process for coaches to become certified, but did discuss ways to help the coaches learn after certification and ensure coaches were maintaining their skills and knowledge. Suggestions included incorporating a community of practice or forum for coaches to connect with and learn from other coaches; refresher courses to maintain certification; and regular meetings with coaches at each site led by site leads.

--INSERT TABLE 3 HERE--

**Discussion**

E-learning is an efficient and effective way to deliver training to large numbers of people spread across large geographical areas [3,6]. Understanding the perspectives of e-learning training users is important to further refine e-learning training to enhance effectiveness, acceptability, and sustainability. The purpose of this study was to understand [PROGRAM NAME BLINDED FOR REVIEW] coaches’ and staffs’ perspectives on the [PROGRAM NAME BLINDED FOR REVIEW] coach e-learning training. Several themes were constructed in this study to capture coaches’ and staffs’ various perspectives. There were seven main themes constructed from the coach interviews: (a) training design, (b) “I didn’t know what to expect from the training”, (c), technology usability, (d) learning, (e) coaches’ background shaped their training experience, (f) support, and (g) coaches valued the training; and two themes constructed from the staff interviews: (a) streamlining the training delivery and (b) ensuring coaches meet the “[PROGRAM NAME BLINDED FOR REVIEW] standard”.

The online modules and resource centre were favourably viewed by all coaches and staff. Technology issues are often cited as one of the downsides to e-learning [3], however, it is possible that the high up-front cost associated with the online platform for this training helped to avoid technology issues. Staff suggested additional applications for the online platform to further alleviate their workload required per coach. Automating staff tasks could enhance the cost-effectiveness of the training because once they are implemented, these components would require minimal staff attention and oversight.

Coaches and staff all saw high value in the mock session training component as a means for learning and ensuring coaches were at the [PROGRAM NAME BLINDED FOR REVIEW] standard of delivery before delivering the program to clients at sites. The value placed on the opportunity to apply the learning from the asynchronous modules is echoed in a study by Jones and colleagues [25], where a group of physiotherapists using e-learning found a mock scenario of high value. The nervousness that coaches felt before engaging in the mock session could potentially be minimized by staff suggestions of clearer communication of expectations and better preparing coaches for the mock session. Some coaches will likely feel nervous before the mock session regardless of changes because the purpose of the mock session is to evaluate the coach's MI skills to ensure they meet the [PROGRAM NAME BLINDED FOR REVIEW] standard to pass the training. These feelings of nervousness related to a mock scenario were also identified in the study of physiotherapists [25]. Coaches’ desire for more synchronous and in-person training components, specifically surrounding the mock session (see *coach subtheme 1.4 training synchronicity and mode*) needs to be balanced with sustainability as the training scales up and reaches more coaches. The staff recommendation of promoting coaches practice for the mock session with peers and the site lead, has the possibility of striking the balance between giving coaches further practice and maintaining a sustainable training process. It is possible that as e-learning continues to become more widespread, coaches will have less desire for in-person and synchronous components due to their familiarity and confidence in e-learning training designs and their ability to learn through e-learning training.

The majority of the [PROGRAM NAME BLINDED FOR REVIEW] coach e-learning training design and development efforts were focussed on the process coaches would go through to become certified [13]. Less attention was paid to the staff delivery of the e-learning training, which was reflected in several themes. Standardization of staff procedures, including clearer communication, needs to be highly prioritized to enhance both the coaches’ and staffs’ training experiences. It is possible that less attention was given to the staff experience due to a scarcity of research on e-learning training design and development [7].

Evaluating user experience for both those taking and delivering e-learning training is important to capture nuances associated with training design. Despite the training design still needing further improvements, it is promising that coaches highlighted that they learned from the [PROGRAM NAME BLINDED FOR REVIEW] coach e-learning training. Coaches shared what they learned and discussed their ability to practice and apply their gained knowledge and skills both within their [PROGRAM NAME BLINDED FOR REVIEW] sessions and other contexts. The training design allowed coaches to follow a systematic training process, while also utilizing their own learning strategies and approaches. One strength to e-learning over in-person training is that learners can utilize their own learning strategies and to rewatch e-learning modules at their convenience. [PROGRAM NAME BLINDED FOR REVIEW] coaches highlighted this as a strength, and this has also been cited as a strength in research involving healthcare students [26]. Incorporating input from [PROGRAM NAME BLINDED FOR REVIEW] coaches during the design and development phases of the [PROGRAM NAME BLINDED FOR REVIEW] coach e-learning training and working with experts in e-learning likely played a role in attenuating different learning styles.

Our findings support Cook and Ellaway’s [7] sentiment of the importance of assessing participant experience and satisfaction. Despite thoroughly incorporating [PROGRAM NAME BLINDED FOR REVIEW] coach input and feedback during the design and development processes of the [PROGRAM NAME BLINDED FOR REVIEW] coach e-learning training, there are still many areas for improvement within the training. The two main user groups for this e-learning training were [PROGRAM NAME BLINDED FOR REVIEW] coaches and the [PROGRAM NAME BLINDED FOR REVIEW] staff facilitating the delivery of the training. Findings suggest that investing both time and resources into usable and high-quality technology may be worthwhile to enhance user experience. The use of technology that goes beyond PowerPoint slides and voice-over may lead to improved learner engagement as well as reducing staff burden.

Based on the feedback provided in this study from both coaches and training delivery staff, the [PROGRAM NAME BLINDED FOR REVIEW] coach e-learning training was modified. Modifications included automating more training steps to reduce staff burden, delivery staff providing more and clearer with coaches regarding training expectations, and modifying and adding coach resources to the resource centre. With more fitness and recreation facilities being onboarded to deliver [PROGRAM NAME BLINDED FOR REVIEW], the coach e-learning training is currently scaling up. The modifications that were made to the training gives us confidence that the timing of the training scale-up is appropriate.

The results of this paper have led us to develop the following recommendations for individuals and groups developing their own TEL within the health context:

1. Invest in resources (i.e., time, money) during the development stages of e-learning because learners value high quality training.
2. Provide learners the opportunity to gain knowledge and then practice skills.
3. Providing feedback on learners’ progress is an additional mode of learning.
4. Ensure clear communication to learners is provided throughout the training process.
5. If e-learning includes asynchronous components, suggesting that learners study or practice together could be beneficial.
6. Standardize and automate as many steps of the e-learning process as possible to ensure an efficient process for learners and staff.

*Limitations*

The sample of coaches and staff was fairly homogenous with regard to the age of staff, which could have implications regarding technology literacy. As [PROGRAM NAME BLINDED FOR REVIEW] continues to scale-up nationally and globally, there will be greater diversity in coach and staff demographics, and future research may be needed to further explore the experiences of those belonging to demographic groups not represented in this study. Secondly, this study was specific to the [PROGRAM NAME BLINDED FOR REVIEW] coach e-learning training, which limits generalizability of study results. However, we attempted to structure the coding templates and results in a manner that other e-learning training developers or evaluators could learn from these results. Additionally, coach interviews were predominantly conducted approximately three months after certification, which could limit their memory of how long they took to complete training and could have affected their perspective on the training. While we attempted to minimize power dynamics between interviewers and participants, it is possible that participants perceived power differentials, which could have affected the data. Furthermore, as with all research, it is possible that participants were not honest or had reservations about providing negative feedback about the [PROGRAM NAME BLINDED FOR REVIEW] coach e-learning training. We attempted to reduce the chances of this by asking participants for all positive and negative comments and assuring participants that negative feedback would not affect their roles in [PROGRAM NAME BLINDED FOR REVIEW]. We believe this helped with participant honesty, as we did receive feedback from participants on areas for improvement within the e-learning training.

*Future directions*

Coaches and staff discussed that coaches learned from the [PROGRAM NAME BLINDED FOR REVIEW] e-learning training and that there was a positive trickle-down effect for clients. In line with Cook and Ellaway’s TEL evaluation framework [7], future research must assess the learning outcomes (e.g., Kirkpatrick levels 2, 3, and 4) from this training, and a cost estimate of this training should be conducted to fully understand the sustainability.

**Conclusions**

This study highlights the perspectives and experiences of coaches and staff as they interacted with the [PROGRAM NAME BLINDED FOR REVIEW] coach e-learning training. Understanding coach and staff perspectives is important given that they are the individuals delivering and receiving the training. Results demonstrated that coaches and staff valued a high-quality online platform, the importance of developing training with both coaches and staff in mind, and that incorporating several learning modalities and modes could cater to different learning styles and backgrounds. E-learning developers can consider the results from this study when creating their own e-learning platforms to enhance user satisfaction. This study demonstrates that continued evaluation beyond the design and development of e-learning training can provide insightful information which could lead to more effective, efficient, and acceptable e-learning training iterations.

**Abbreviations:**

IKT: integrated knowledge translation

MI: motivational interviewing

NDA: non-disclosure agreement

TEL: technology-enhanced learning

**Declarations**

**Ethics approval and consent to participate**

Ethical approval was obtained from the [INSTITUTION NAME BLINDED FOR REVIEW] Behavioural Research Ethics Board (H21-01800). Informed consent was obtained from all individual participants included in the study.

**Consent for publication**

Not applicable.

**Availability of data and materials**

The dataset analyzed during the current study are available from the corresponding author on reasonable request.

**Competing interests**

The authors declare that they have no competing interests.

**Funding**

This research was funded by both a Social Sciences and Humanities Research Council Doctoral Scholarship and a Canadian Institutes of health Research grant.

**Authors’ contributions**

KDC, NJG, and MEJ contributed to the conceptualization, design, writing, and revisions of the manuscript. KDC and NJG conducted data collection and data analysis. KDC completed the first draft of the manuscript. NJG and MEJ reviewed and approved the final manuscript.

**Acknowledgement**s

Not applicable.

**References**

[1] Roskvist R, Eggleton K, Goodyear-Smith F. Provision of e-learning programmes to replace undergraduate medical students’ clinical general practice attachments during COVID-19 stand-down. Educ Prim Care 2020;31:247–54. https://doi.org/10.1080/14739879.2020.1772123.

[2] Seymour-Walsh A, Bell A, Webber A, Smith T. Adapting to a new reality: COVID-19 coronavirus and online education in the health professions. Rural Remote Health 2020. https://doi.org/10.22605/RRH6000.

[3] Kimura R, Matsunaga M, Barroga E, Hayashi N. Asynchronous e-learning with technology-enabled and enhanced training for continuing education of nurses: a scoping review. BMC Med Educ 2023;23:505. https://doi.org/10.1186/s12909-023-04477-w.

[4] Kulaksız T, Steinbacher J, Kalz M. Technology-Enhanced Learning in the Education of Oncology Medical Professionals: A Systematic Literature Review. J Cancer Educ 2023;38:1743–51. https://doi.org/10.1007/s13187-023-02329-1.

[5] Rouleau G, Gagnon M, Côte J, Payne-Gagnon J, Hudson E, Dubois C, et al. Effects of e-learning in a continuing education context on nursing care: Systematic review of systematic qualitative, quantitative, and mixed-studies reviews. J Med Internet Res 2019;21.

[6] Cook DA. Web-based learning: pros, cons and controversies. Clin Med 2007;7:37–42.

[7] Cook DA, Ellaway RH. Evaluating technology-enhanced learning: A comprehensive framework. Med Teach 2015;37:961–70. https://doi.org/10.3109/0142159X.2015.1009024.

[8] Yardley L, Morrison L, Bradbury K, Muller I. The Person-Based Approach to Intervention Development: Application to Digital Health-Related Behavior Change Interventions. J Med Internet Res 2015;17:e30. https://doi.org/10.2196/jmir.4055.

[9] Yardley L, Ainsworth B, Arden-Close E, Muller I. The person-based approach to enhancing the acceptability and feasibility of interventions. Pilot Feasibility Stud 2015;1:37. https://doi.org/10.1186/s40814-015-0033-z.

[10] Miller WR, Rollnick S. Motivational interviewing: helping people change and grow. Fourth edition. New York: The Guilford Press; 2023.

[11] Jull J, Giles A, Graham ID. Community-based participatory research and integrated knowledge translation: advancing the co-creation of knowledge. Implement Sci 2017;12:150. https://doi.org/10.1186/s13012-017-0696-3.

[12] Gainforth HL, Hoekstra F, McKay R, McBride CB, Sweet SN, Martin Ginis KA, et al. Integrated Knowledge Translation Guiding Principles for Conducting and Disseminating Spinal Cord Injury Research in Partnership. Arch Phys Med Rehabil 2021;102:656–63. https://doi.org/10.1016/j.apmr.2020.09.393.

[13] Cranston KD, Grieve NJ, Dineen TE, Jung ME. Designing and Developing Online Training for Diabetes Prevention Program Coaches Using an Integrated Knowledge Translation Approach: Development and Usability Study. JMIR Form Res 2024;8:e50942. https://doi.org/10.2196/50942.

[14] Jackson C, Butterworth S, Hall A, Gilbert J. Motivational Interviewing Competency Assessment (MICA) 2015.

[15] Grieve N, Cranston KD, Jung ME. Examining the effectiveness of an e-learning training course for coaches of a type 2 diabetes prevention program. J Technol Behav Sci 2023:1–11.

[16] James W. Pragmatism’s conception of truth. J Philos Psychol Sci Methods 1907;4:141–55.

[17] Kaushik V, Walsh CA. Pragmatism as a Research Paradigm and Its Implications for Social Work Research. Soc Sci 2019;8:255. https://doi.org/10.3390/socsci8090255.

[18] Sandelowski M. Whatever happened to qualitative description? Res Nurs Health 2000;23:334–40.

[19] Tracy SJ. Qualitative Quality: Eight “Big-Tent” Criteria for Excellent Qualitative Research. Qual Inq 2010;16:837–51. https://doi.org/10.1177/1077800410383121.

[20] Smith B, McGannon KR. Developing rigor in qualitative research: problems and opportunities within sport and exercise psychology. Int Rev Sport Exerc Psychol 2018;11:101–21. https://doi.org/10.1080/1750984X.2017.1317357.

[21] Sparkes AC, Smith B. Judging the quality of qualitative inquiry: Criteriology and relativism in action. Psychol Sport Exerc 2009;10:491–7. https://doi.org/10.1016/j.psychsport.2009.02.006.

[22] Malterud K, Siersma VD, Guassora AD. Sample Size in Qualitative Interview Studies: Guided by Information Power. Qual Health Res 2016;26:1753–60. https://doi.org/10.1177/1049732315617444.

[23] King N. Using templates in the thematic analysis of text. Essent. Guide Qual. Methods Organ. Res., Sage Publications; 2004, p. 256.

[24] Brooks J, McCluskey S, Turley E, King N. The Utility of Template Analysis in Qualitative Psychology Research. Qual Res Psychol 2015;12:202–22. https://doi.org/10.1080/14780887.2014.955224.

[25] Jones SE, Campbell PK, Kimp AJ, Bennell K, Foster NE, Russell T, et al. Evaluation of a Novel e-Learning Program for Physiotherapists to Manage Knee Osteoarthritis via Telehealth: Qualitative Study Nested in the PEAK (Physiotherapy Exercise and Physical Activity for Knee Osteoarthritis) Randomized Controlled Trial. J Med Internet Res 2021;23:e25872. https://doi.org/10.2196/25872.

[26] Liao F, Murphy D, Wu J-C, Chen C-Y, Chang C-C, Tsai P-F. How technology-enhanced experiential e-learning can facilitate the development of person-centred communication skills online for health-care students: a qualitative study. BMC Med Educ 2022;22:60. https://doi.org/10.1186/s12909-022-03127-x.

Table 2. Coach interview themes and exemplar quotes

| Themes | Exemplar quotes |
| --- | --- |
| 1 Training design | n/a |
| 1.1 Course content was comprehensive | “There’s quite a bit of training and it’s very in-depth training.” - Tina |
| 1.2 A variety of learning modalities | “I would say [the mock session] was really helpful because, yeah, it kind of gets the kinks out before you actually do it [with real clients].” - Deborah |
| 1.3 Online platform organization | “[The resource centre was] labelled well enough so you know what you’re looking for or looking at.” - Tina |
| 1.4 Training synchronicity and mode: asynchronous vs. synchronous | “[I] learn better when a human talks to me instead of [watching a] video.” - Lebron |
| 1.5 Training process | n/a |
| 1.5.1 Communication to coaches needs to improve | “[It would be nice to have] a simple graph that says, you know, first step is a pre-test, and then the seven modules with quizzes.” - Marisol |
| 1.5.2 Systematic steps to becoming a certified coach | “[The training followed] a competency-based approach, which…was enlightening…and a complete process because there was a pretest for the knowledge, then it went into the modules, and then it went to the mock…So theoretically, do you know the information, now you can apply it and implement…and then a final test for knowledge.” - Marisol |
| 1.6 Coach testing is necessary | “I obviously took [the mock session] pretty seriously and wanted to pass it.” - Marisol |
| 1.7 Receiving feedback is useful | “It was good because there were some things that I missed [in the mock session] and that was pointed out to me. So, it was good, once again, going into my first session to remind myself not to forget certain items.” - Mike |
| 2 “I didn’t know what to expect from the training” | “[I knew that I’d learn about] prediabetes and helping clients to know how to exercise and to eat healthier.” - Sally |
| 3 Satisfaction with technology usability | “Everything worked as it should, which is good because I’m pretty technologically challenged. So didn’t have any issues.” - Tina |
| 4 Learning | n/a |
| 4.1 Coaches learned | n/a |
| 4.1.1 Learning new knowledge | “[I learned that] it comes down to more obviously client-centred…more or less kind of motivate them.” - Paul |
| 4.1.2 Demonstrating new knowledge | “[I learned] the skills on like coaching things, like how to concisely teach the talk test, and how to concisely talk about carbohydrates and sugar. And only like giving the actual information that is wanted from the client, so not word vomiting a bunch of information to people, and so just being like, oh, what do they want…kind of listening.” - Deborah |
| 4.1.3 Readiness to deliver SSBC sessions | “The actual modules and the training process, whether it was like the pretest and the modules right through the mock session, I felt competent at the end – that I can start to go and deliver to the clients.” - Marisol |
| 4.1.4 Application of learning | n/a |
| 4.1.4.1 Anticipating application of learning within SSBC | “I have to be careful of the biases, right. So like, I can walk into a gym and I’m not intimidated, right. But I think for some people that will be their first experience, right? Or it could be very intimidating. So I think there’s some things that we have in our, you know, in our own toolbox that will apply and make us more aware.” - Marisol |
| 4.1.4.2 Readiness to apply learning within SSBC | “I think the most important…the things that I can see that I changed about the way I talk to clients, that I do differently in the [PROGRAM NAME BLINDED FOR REVIEW] is listen more. So ask them to tell more about things instead of just providing them [with information].” - Sally |
| 4.1.4.3 Transcending SSBC – applying learning in other contexts | “I do find the motivational interviewing can actually be very helpful in the personal training.” - Tina |
| 4.2 The learning journey | n/a |
| 4.2.1 Individual learning strategies | “I took notes, and I’m old school, so I write things down. Right. And I [used] cue cards as well, for when I was preparing for the mock, and also for delivering the sessions with the client.” - Marisol |
| 4.2.2 Gradual learning curve | “I assumed [the training] was going to be easier than it was. So it was a learning curve.” - Mike |
| 4.2.3 “Practice, practice, practice” before the mock session | “I started to practice [MI] around the house with my husband and friends and family.” - Marisol |
| 4.2.4 The nerve-wracking experience of being evaluated | “[The [PROGRAM NAME BLINDED FOR REVIEW] staff] guided me through in the beginning of the session so that I knew what to, like, expect, which helped me a lot to relax a little bit and focus more on what I had to ask and accomplish.” - Ryan |
| 4.2.5 Learning through trial and error | “[My colleague failed the mock session.] So I asked, what happened? What didn’t you do?...[They] showed me the printouts and how to go over it…It definitely helped that my co-worker did [the training] before me.” - Lebron |
| 4.2.6 Continued learning after certification | n/a |
| 4.2.6.1 Using resources to foster session success | “With my first two clients, I mentioned to them that they were my first two clients. So I had the [guide] in front of me. So I was making sure that I [had] all the information down. And I even told them like, I don’t want to be like robotic, but I’m just like learning.” - Ryan |
| 4.2.6.2 Real-world practice enhances mastery | “I think [with] every client, I’m a little bit better at [using MI].” - Tina |
| 4.2.6.3 Supplemental learning ideas | “It’s easy to swing back to our old, I guess, ways of doing things as a coach.” - Marisol |
| 5 Coaches’ backgrounds shaped their training experience | “The motivational interview, that is something completely new for me. I’m way better than before the training, but I still think I have a lot to improve on…And for the specifically about diabetes. I already knew those information. So that thing wasn’t new for me. And it’s a good part because then I can focus my efforts in being better about how to coach the session and not necessarily about to know the technical information.” - Sally |
| 6 Support | n/a |
| 6.1 [PROGRAM NAME BLINDED FOR REVIEW] staff-to-coach support | “The feedback was really nice and encouraging and gave helpful tips and stuff.” - Deborah |
| 6.2 Coach-to-coach support | “I printed [the resources] off for all my coaches that are coming…so they have a successful first, first interview.” - Tina |
| 7 Coaches valued the training | n/a |
| 7.1 Viewing the training as continuing education | “Especially for personal trainers, I think we do learn a lot about exercising, but we don’t learn about how to talk to people. So it’s not just telling them that they should exercise and that they will get the importance of it. So it is very important for us to know more [about] how to talk to people, and the [PROGRAM NAME BLINDED FOR REVIEW] training helps us to do this.” - Sally |
| 7.2 The trickle-down effect of coach training on clients | “As I get better at [MI], I think it is helping [my clients] take a bigger role in how they’re coming up with the ideas themselves. And I think that in the long run, they will be more successful than me telling them what to do.” - Tina |
| 7.3 Extending the training to non-coaching staff | “I think it would be really good for like all the managers to do motivational interviewing, even customer service and aquatics. As well as the inclusivity, I think it’s important. I don’t think every department has to learn about diabetes prevention, but I think just some of the like, skills are transferrable.” - Deborah |

n/a: no exemplar quotes for themes containing lower order themes.

Table 3. Staff interview themes and exemplar quotes

| Themes | Exemplar quote |
| --- | --- |
| 1 Streamlining the training delivery | n/a |
| 1.1 What’s working | n/a |
| 1.1.1 The online platform usability | “I really like the way that the platform kind of takes control of the training for most of it. And then there’s only really three instances where I need to be in contact with them. And the like, there seems to be a kind of flexibility around what notifications I can send from the platform, which is quite useful.” - John |
| 1.1.2 Staff communication isn’t all bad | “[The staff are] supporting [coaches] throughout the process…even though we’re getting a lot of emails, John’s always responding. So they should feel hopefully supported.” - Jennifer |
| 1.1.3 On the way to a sustainable process | “[The online training] process has allowed us to train a lot of coaches really quickly with less resources [than in-person training], and I think that’s a huge win.” - Jennifer |
| 1.2 Removing unnecessary training steps and standardizing procedures | “And something I’ve been doing is to just whenever I release a notification [from the platform], I also email [site leads], particularly at the start because if it gets redirected to their spam folder then they can move it and [their inbox] should accept the rest of the emails that come. But I have wondered if it’s maybe worth like, at the start of new sites, asking site leads or senior management to whitelist the emails from [the online platform] so they’re never blocked.” - John |
| 1.3 Redistributing workload | n/a |
| 1.3.1 Automating procedures | “It’s a bit like administratively laborious to have the NDA separate when it could be included in the [online platform] login page.” - John |
| 1.3.2 Defined roles | “[There is a] back and forth between a coach and the like three different people on our team….it feels like there’s so many spots where something can get messed up.” - Sophie |
| 1.4 Clearly communicating all expectations | “Maybe [providing] just a bit more information about what the mock session is…and possibly like, the fact that it takes 45 minutes.” - John |
| 2. Ensuring coaches meet the “[PROGRAM NAME BLINDED FOR REVIEW] standard” | n/a |
| 2.1 Extra support for the first coach at each site | “The mock session can feel a bit intimidating, particularly for people who are either the first in their site or just haven’t had contact with anyone else in the site. And it’s come up [in conversations with coaches] that support around the mock would be valuable.” - John |
| 2.2 Setting coaches up for mock session success | n/a |
| 2.2.1 More realistic mock sessions | “95% of the mock sessions I code is the coach with the script open on their desk, reading it line for line for line for line. And if we want our coaches to be delivering [PROGRAM NAME BLINDED FOR REVIEW] at a client-centred way, having conversations their client, reading a script isn’t doing that.” - Sophie |
| 2.2.2 Resource modification | “I just sometimes think that the script, like the script for like, the sessions are actually like, confusing a little. I feel like there’s a lot of information in them so then [coaches]…we’re trying to make it as like simple as possible by giving them all this info, but it’s actually really overwhelming.” - Sophie |
| 2.2.3 Encouraging practice | “Adding another mock session is not sustainable. I think encouraging potential practice though, with especially at the site…Maybe we upload like a mock client script of some sort [to the resource centre], and they can like practice with someone at their [site] who’s also training and they can switch roles.” - Jennifer |
| 2.3 Feedback is a necessary training component | “I think the feedback is almost equally [important as doing the mock session]…I write them feedback so that if they were to implement all of those pieces [from the feedback], they would be delivering [PROGRAM NAME BLINDED FOR REVIEW] [to our standards].” - Sophie |
| 2.4 Importance of testing coaches | “[Coaches] learn a lot from the modules. I think a mock session gives them a chance to practice their skills and get feedback.” - Jennifer |
| 2.5 Can’t stop training after certification | “It think it could be beneficial for a yearly, two-hour Zoom booster, where there’s a 40-minute refresher – what MI is, maybe some mock scenarios with [the training delivery staff]. And [coaches] can tune in, watch it, we record it, or send it to them. And maybe there’s some practice breakout rooms, something like that.” - Jennifer |

n/a: no exemplar quotes for themes containing lower order themes.
